# Analysis of gastric mucosa associated microbiota in functional dyspepsia using 16S rRNA gene next-generation sequencing

**DOI:** 10.1186/s12866-025-04095-0

**Published:** 2025-06-26

**Authors:** Noha Salah Soliman, May Sherif Soliman, Walied Elhossary, Amani Ali El-Kholy

**Affiliations:** 1 Clinical and Chemical Pathology, Faculty of Medicine, Cairo University, Cairo, Egypt; 2 Clinical and Chemical Pathology, Faculty of Medicine, Cairo University, Cairo, Egypt; 3 Endemic Diseases and Gastroenterology, Faculty of Medicine, Cairo University, Cairo, Egypt

**Keywords:** Gastric biopsies, Microbiota, Functional dyspepsia, 16S rRNA gene NGS

## Abstract

Gastric disorders have been associated with changes in the abundance and composition of microbiota, which typically coexist in symbiosis within the stomach. There is a scarcity of data regarding the presence of gastric mucosa-associated dysbiosis in functional dyspepsia. The present study aimed to characterize the taxonomy and diversities of the microbiota in the gastric mucosa of patients with functional dyspepsia. The study was conducted on a total of 50 paired antral and body gastric biopsies collected from the dyspepsia group (*n* = 15) and control group (*n* = 10). Microbial DNA was extracted from all gastric biopsies, followed by 16 S rRNA gene next-generation sequencing (NGS) using the Miseq Illumina platform. Firmicutes, Proteobacteria, and Bacteroides were the most predominant phyla in both groups, with a significant overrepresentation of Proteobacteria in the dyspepsia group (*p* value = 0.004). The genera *Streptococcus*, *Prevotella*, and *Helicobacter* were the most prevalent in both groups. The species of *H. pylori* and *Prevotella melaninogenica* were significantly more abundant in the dyspepsia group. The species of The LEfSe analysis revealed that *E. coli*, *Helicobacter*, *Pseudomonas*, *Bifidobacteria*, and *Enterobacteriaceae* were the most highly abundant and discriminating taxa in the gastric biopsies of the dyspepsia group compared to the control group. The microbial alpha diversity was significantly higher among gastric biopsies of dyspepsia than controls (*P* = 0.031). The beta diversity showed microbial dissimilarity between samples of dyspepsia and the control group. The 16 S rRNA gene NGS used in the present study demonstrated significant alteration in composition and diversities of gastric mucosa-associated microbiota among cases of functional dyspepsia compared to the controls. It is advisable to utilize advanced innovative technologies to gain a deeper understanding of the underlying pathophysiology of disrupted microbiota in gastric disorders.

**Supplementary Information:**

The online version contains supplementary material available at 10.1186/s12866-025-04095-0.

## Introduction

Dyspepsia is a distressing gut disorder that affects a considerable size of the population. Published data has reported cases of dyspepsia at geographically variable estimated rates of 10–40% [1]. Patients with dyspepsia may suffer clinically from discomforting upper gastrointestinal symptoms that disturb their healthy life style. The etiology of structural dyspepsia can be explained by the presence of organic causes in stomach, such as gastric inflammation, ulceration and oesophageal reflux. However, unfortunately, the majority dyspepsia cases may be overlooked, due the absence of specific detectable organic cause posing a clinical diagnostic challenge [2]. Dyspepsia can be influenced by an interplay of an array of underlying pedisposig factors, mostly related to dietary habits that may differ across populations having geographical and ethnic variations [3].

Human gut microbiome has been lately, regarded as a pivotal player in several health disorders and can comprise a non-inferior role in dyspepsia [4]. The dynamic nature of microbial composition in stomach and gut is controlld by several demographic, genetic factors, as well as dietary habits and medications drug- intake [5]. The gut microbiome, host immunity, and biological systems engage in reciprocal communication. In normal conditions, the microbiota protects against various diseases by regulating the immune system [4]. In cases of illness, the disturbance of a balanced microbiota (referred to as dysbiosis) can trigger immune dysfunction, resulting in detrimental pro-inflammatory effects [4, 6]. Numerous studies have established a correlation between dysbiosis, an alteration in the composition of the stomach microbiota, and a variety of gastric disorders ranging from mild to severe gastric neoplasms [3].

The examination of the human microbiome is considered challenging due to the complexity of the existing microbial communities. Moreover, studying healthy microbiota is hindered by the dynamic variability of microbial composition across individuals, which is influenced by several associated factors [4]. Traditional approaches to investigating the gastric microbiota involved the utilization of traditional microbiological methods such as culture and biochemical identification. However, these methods fail to identify a considerable proportion of uncultivable organisms [7]. Recently, the field of molecular technologies has witnessed significant progress by tangible development in proteomics, metagenomics, and whole genome sequencing (WGS), which have facilitated the comprehensive characterization of microbiota, that can potentially enhance our knowledge of gastric microbiota in both normal conditions and medical disorders [3]. Since the colon contains the most significant number of microbial populations, it has been the subject of the majority of human gut microbe research compared to the stomach [8]. Limited research has been performed in Egypt regarding the human gastrointestinal microbiota and the potential relatedness to a variety of health disorders, primarily due to financial and technical constraints [9]. In particular, the characterization of stomach microbiota from gastric biopsies has not been extensively investigated in Egypt.

Therefore, we aimed to identify alterations in gastric mucosa-associated microbiota composition and diversities among patients with functional dyspepsia using 16 S rRNA gene Next Generation Sequencing (NGS) microbiome profiling technology.

## Methodology

### Human subjects and specimen collection

The present study was carried out using gastric biopsy samples from patients clinically indicated for endoscopy among a scheduled list of routine upper endoscopies conducted in Kasr-AlAiny Cairo University hospital. Paired antrum and body gastric biopsies were collected from a total of 25 participants allocated into 2 groups: the control group (*n* = 10) and functional dyspepsia group (*n* = 15). The classification of cases of functional dyspepsia was made according to the Rome III classification [10]. The control group included patients not fulfilling criteria of functional dyspepsia who were eligible for endoscopy for reasons other than dyspeptic symptoms, such as oesophageal varices or investigating anaemia [11]. Our study excluded patients with a recent intake of antibiotics, probiotics, proton pump inhibitors and those with history of reflux esophagitis, gastroduodenal ulcers, or gastrointestinal surgery [12].

All enrolled human subjects underwent upper gastrointestinal endoscopy according to safe standard procedures. During endoscopy, routine mucosal tissue biopsies were taken from the antral and body areas of the stomach. Subsequently, specimens were transported in cryovial tubes prefilled with sterile thioglycolate or glycerol broth and then stored at −80C° for further molecular investigations.

### Ethical statement

An ethical approval was granted from the Research Ethics Committee at the Faculty of Medicine, Cairo University, approval number N-497–2023. The study was conducted in adherence to the principles of of the Helsinki Declaration, and all participants wrote an informed consent.

### DNA extraction and 16 S rRNA gene sequencing

Gastric biopsies were homogenized with a phosphate buffer, and then the DNA was extracted using the QIAamp tissue DNA Mini Kit (Qiagen, USA) following the prescribed manufacturer’s instructions. The gene sequence primers specifically focused on amplifying the V3 and V4 regions using the 16 S PCR Amplicon Forward and Reverse Primers. The Sequencing Library was set in adherence to Illumina protocol (Illumina, USA) [13]. The KAPA HiFi HotStart Ready Mix (Kapa Biosystems, USA) was used for PCR with 35 amplification cycles at 60 °C annealing temperature. The Agencourt AMPure XP Kit (Beckman Coulter, Japan) was used to purify amplicons, which then were tagged with the indexes of the Nextera XT Kit (Illumina, USA). Libraries were normalized to 4nM, mixed with PhiX Control 5% Kit v3 (Illumina, USA) and denatured following the manufacturer’s guidelines. MiSeq sequencing was carried out using the MiSeq Illumina Reagent Kit v3 MS-102–3003 (600-cycle format; Illumina) [14].

### Processing of sequencing data and group comparisons

The raw data generated from 16S rRNA gene sequencing was subjected to quality filtering followed by Rarefaction analysis to evaluate sequencing depth. Further analysis was done, including taxonomic classification, clustering, and annotation of operation taxonomic units (OTU) by uploading fastaQ files on Ezbiocloud software program (EzBiome, Inc, USA) [15]. In order to compare microbial taxonomic profiles and diversities, the following comparisons were established: control versus dyspepsia, antrum versus body, and *H. pylori* positive (-pos) versus *H. pylori* negative (-neg) groups. Using the Ez Biocloud software, measures of microbial diversity were evaluated in each group: Shannon index (alpha diversity) and the Bray-Curtis dissimilarity index (beta diversity), with plotted two-dimensional principal coordinate analysis (PcoA). For taxonomic biomarker discovery, linear discriminant analysis effect size (LEfSe) was calculated and presented in a cladogram to demonstrate the taxa with the highest discrimination between compared groups [12].

### Microbiome functional analysis

The functional analysis of gastric microbiota was conducted using the phylogenetic investigation of communities by reconstruction of unobserved states (PICRUSt) to determine the metabolic potentials of microbial communities. Functional pathways and orthologs were inferred based on the resemblance between metagenomic sequences with those in the Kyoto Encyclopedia of Genes and Genomes (KEGG) core databases [16]. 

### Statistical analysis

The qualitative data was represented using numerical values and percentages, while the quantitative data was presented by mean/standard deviation. Tests of significance were conducted using the chi-square test and Mann-Whitney test (for qualitative and quantitative data, respectively. The software utilized the Wilcoxon rank-sum to test significant difference in group comparisons for both microbial abundance and alpha diversity. Furthermore, PERMANOVA (permutational multi-variate analysis-of- variance) was utilized to assess the differences in measures of beta diversity measures between the groups under study. A *p* value < 0.05 is considered significant [17]. To ensure a conservative interpretation of significant difference between comparison groups, adjusted False Dicovery Rate (FDR) was considered.

#### Results

The 16S rRNA microbiome profiling was carried out on a total of 50 gastric biopsies in the form of paired antral and body biopsies taken from the stomach of 2 groups of participants: the control group (*n* = 10) and the dyspepsia group (*n* = 15). A total of 2,830,747 16SrRNA V3-V4 valid sequencing reads have been obtained with an average of 56,614.94 ± 21,176.25 reads (Supplementary Tables 1 and Supplementary Fig. 1). The rarefaction analysis denoted ability to capture a significant portion of the community’s diversity. The estimation of library coverage ranged from 99.32 to 99.97% using Good’s estimator. It indicates that the coverage of valid reads for library sequencing results was > 97% of bacteria and effectively represented the majority of gastric microbiota (Supplementary Fig. 2).

**Fig. 1:**
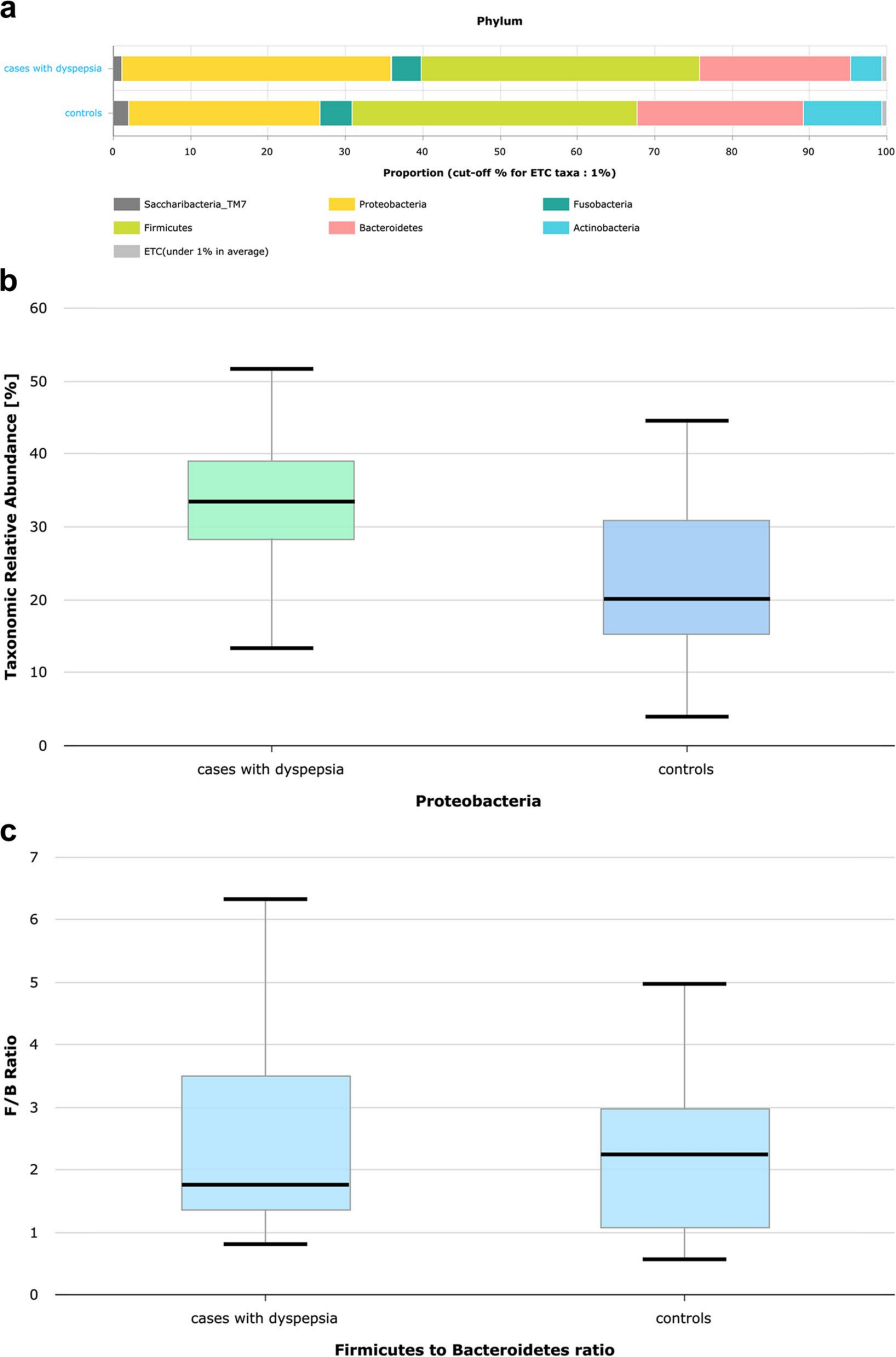
Description of microbial taxonomy (phylum level) in paired gastric biopsies among groups of dyspepsia and controls. **a** Stacked bar showing average relative abundance (RA) of microbial phyla in paired antral and body gastric biopsies of dyspepsia and control groups. **b** Boxplot showing the significantly more enriched average RA of Proteobacteria in gastric biopsies of the dyspepsia group than the control group. The boxes represent interquartile ranges (IQR) with a black line denoting the median and whiskers extending up to the most extremes within 1.5 fold IQR **c**) Boxplot denoting reduced Firmicutes/Bacteroides ratio among the dyspepsia group compared to the control group

The taxonomic analysis of the total sequenced gastric biopsies showed microbiota at the phylum level. The represented taxa had an average relative abundance (RA) > 1.0% (Fig. 1a). The top predominant phyla were observed in the order of Firmicutes, Proteobacteria, and Bacteroidetes, which collectively accounted for less than 65% of the present phyla in each sample, followed by Actinobacteria and Fusobacteria. Spirochaetes were sporadically detected in only 2 samples, which belonged to the phylum with the lowest abundance.

By comparing microbiota composition in dyspepsia and control groups, it was found that paired antrum and body gastric samples of both groups displayed a similar order of predominant phyla. Although Firmicutes and Proteobacteria were the top prevalent phyla in both study groups, there was a significant increase in the abundance of Proteobacteria in the dyspepsia group (*p* value = 0.004), with non-significant lower RA abundance of other phyla (Fig. 1a and b; Table 1). Regarding Firmicutes and Bacteroidetes, which are known to comprise the main components of human gut taxa, a reduced Firmicutes/Bacteroidetes ratio (F/B) was observed among cases of dyspepsia group. However, this difference was not statistically significant (*p* value = 0.563) (Fig. 1c).

**Table 1:** Analysis of microbial taxonomy (phylum level) in gastric biopsies of dyspepsia and control groups

	Control Mean RA (%)	Dyspepsia Mean RA (%)	*p* value
Firmicutes	36.49	36.04	0.835
Proteobacteria	24.44	34.17	0.004
Bacteroidetes	21.93	20.11	0.513
Actinobacteria	10.22	3.92	0.054
Fusobacteria	4.24%	4.01%	0.898

*RA* Relative Abundance, significant *p* value is < 0.05

**Fig. 2:**
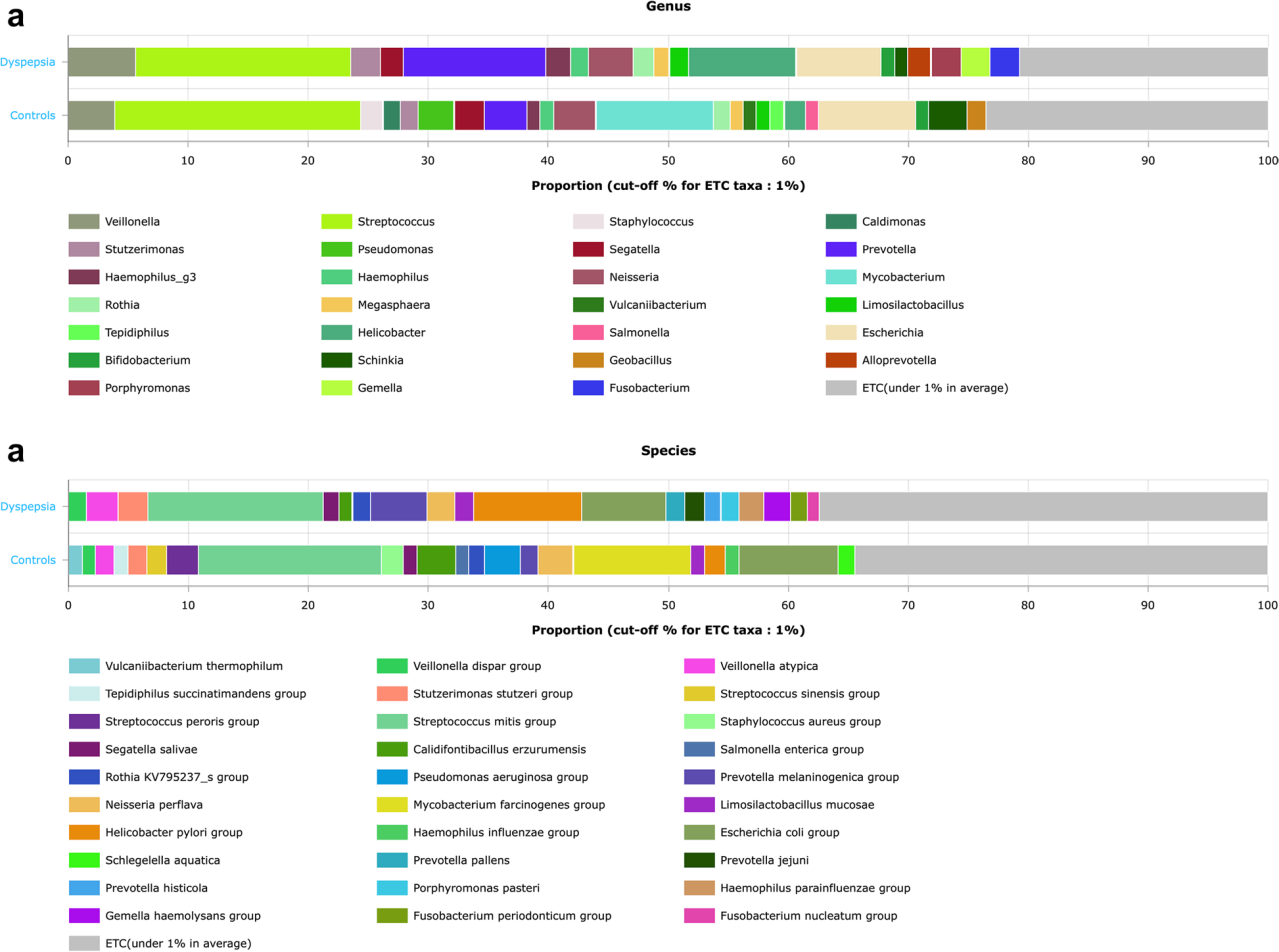
Distribution of microbial taxa among gastric biopsies of dyspepsia cases and controls: **a**) Genus-level analysis, **b** Species-level analysis

**Fig. 3:**
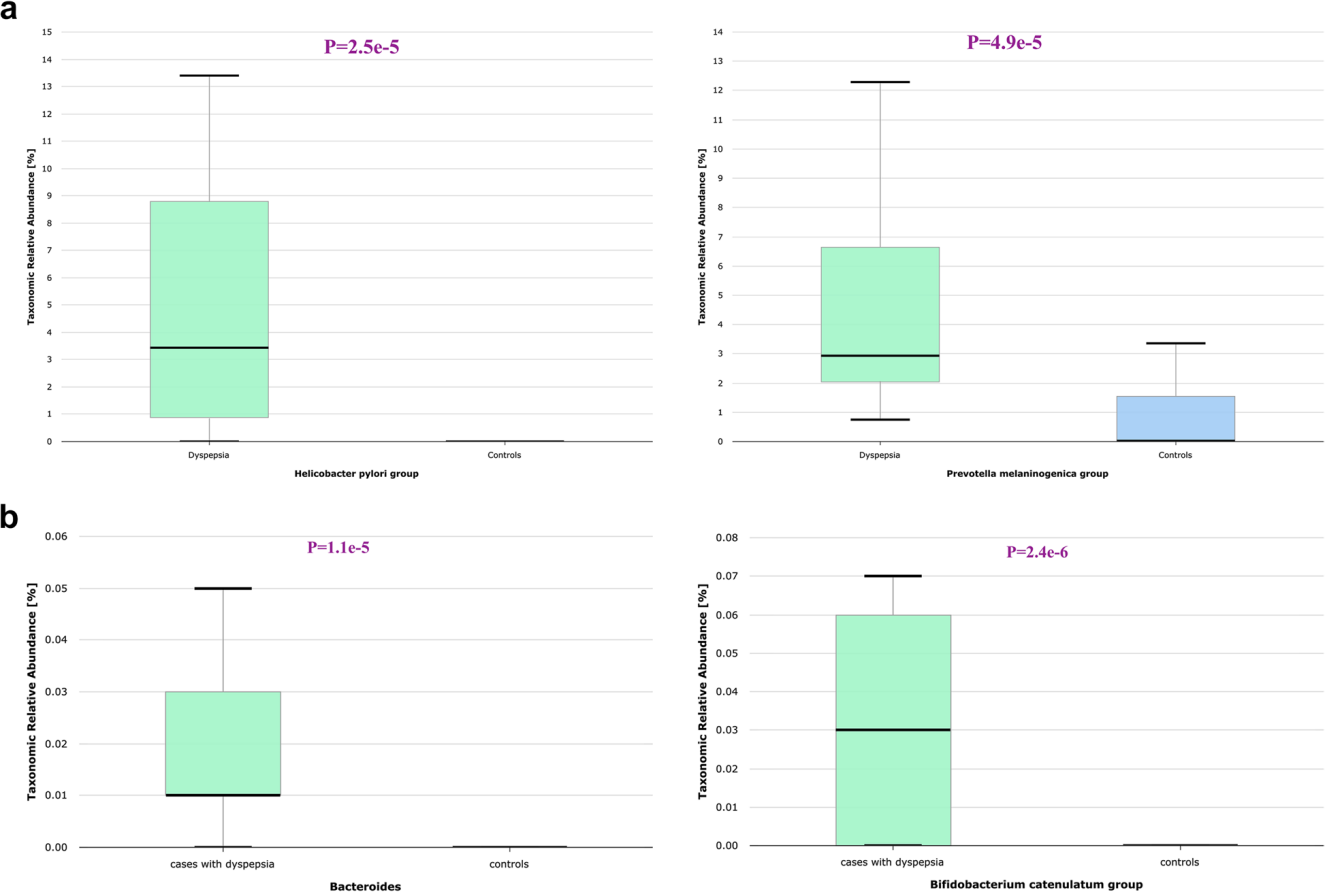
The relevant human gut taxa among cases of dyspepsia compared to controls. **a** Boxplot showing significant overrepresentation of *H. pylori *and *Prevotella melaninogenica group *among cases of dyspepsia compared to controls,** b** Boxplots showing significant over-representation of Bacteroides (left side) and *Bififdobacterium catenulatum* (right side)

At the genus level, the most abundant genera were *Streptococcus* (17.6% in dyspepsia group, 18.12% in control group), *Prevotella* (13.9% in dyspepsia group, 13.3% in control group), *Helicobacter* (10% in dyspepsia group, 7% in control group), and *Veillonella* (5.8% in dyspepsia group, 7.4% in control group) (Fig. 2a). Among these genera, only *Helicobacter* was significantly more represented in the dyspepsia group than the control group (*p* value = 0.029). *Streptococcus* and *Veillonella* belong to the phylum Firmicutes, while *Prevotella* and *Helicobacter* are categorized under the phyla Bacteroidetes and Proteobacteria, respectively. Other genera with lower total RA were identified in the form of *Neisseria* 4.5%, *Rothia* 2.6%, *Lactobacillus* 2.5%, *Haemophilus* 3.9%, *Gemella* 2.2%, and Fusobacteria 2.4%. These genera had similar relative abundances in both the control and dyspepsia groups. At the species level, *Streptococcus mitis*,* H. pylori*, *E. coli* and *Prevotella melaninogenica* were the top predominant species in the dyspepsia group (Fig. 2b), with significant higher abundance observed for *H. pylori* and *Prevotella melaninogenica* compared to the control group (Fig. 3a).

Assessing the microbiota at different gastric anatomical sites showed that in both groups of dyspepsia and controls, antral and body gastric biopsies were comparable in terms of the distribution and RA of the identified taxa in the antral and body gastric biopsies. In the control group, no considerable changes were noted in the RA of Proteobacteria, Bacteroidetes, and Saccharibacteria in body biopsies compared to antral biopsies. However, the other phyla either had lower abundance or had similar abundance levels (Supplementary Fig. 3).

The study identified several significant human gut taxa with relative abundance > 1%, as displayed in Supplementary Fig. 4a. Notably, the dyspepsia group exhibited a higher RA of Proteobacteria (*p* value = 0.002) and Enterobacteriaceae (*p* value = 2.0e-8), than the control group. Certain types of human gut taxa, such as Clostridia (phylum: Firmicutes) and Bacteroides (phylum: Bacteroidetes), were detected at low RA < 1.0%, however both taxa were significantly enriched among samples of the dyspepsia group (*p* value = 0.029), compared to those of the control group (*p* value = 7.6e-6) (Fig. 3b).

Various types of lactic acid bacteria or lactate producing bacteria, commonly known as probiotics, were found in gastric biopsies from both the control and dyspepsia groups. The dominant genera were *Lactobacillus* and *Bifidobacterium*, which are assigned to the Firmicutes and Actinobacteria phyla, respectively, as shown in Supplementary Fig. 4b. The species *Lactobacillus mucosae*, *Bifidobacterium longum*, *Bifidobacterium* breve, and *Bifidobacterium catanulatum* were more enriched in gastric biopsies of dyspepsia cases than controls (Fig. 3b). Although these species are relatively low in abundance, their representation was statistically significant. In contrast, *Weissella confusa* (Phylum: Firmicutes) was more prevalent among controls than dyspepsia cases, with non-statistically significant differences.

**Fig. 4:**
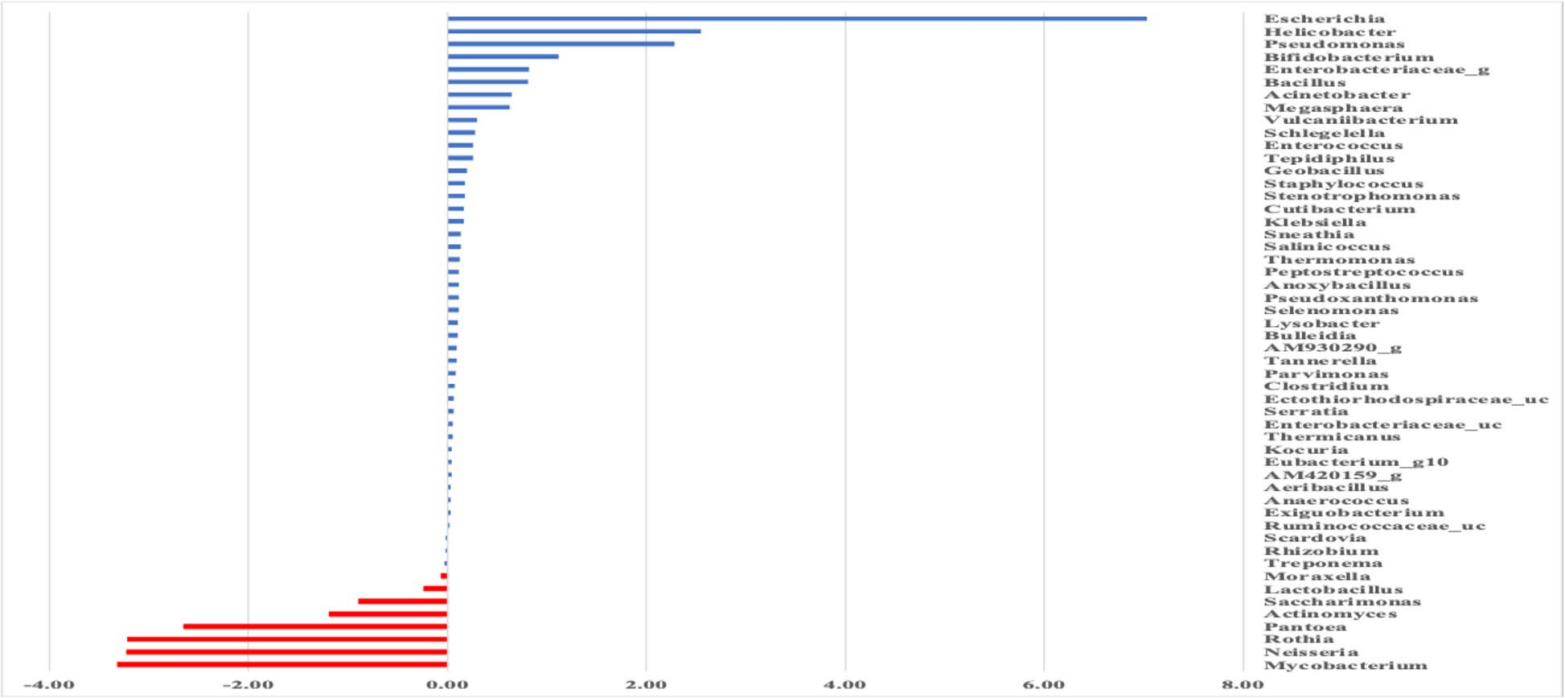
The relevant discriminatory microbial taxa between the control and dyspepsia groups. Linear discriminate analysis effect size (LEfSe) plotted in a cladogram of the relevant discriminatory taxa (genus rank) between the control and dyspepsia groups. The microbial taxa are sorted according to their logarithmic (log10) LDA score. The dyspepsia group is shaded in (blue color), while the control group is in (red color)

The LEfSe analysis identified the discriminative relevant taxa between the dyspepsia and control groups (Fig. 4). In the gastric biopsies of the dyspepsia group, the following taxa were found to be significantly more prevalent: *Escherichia*, *Helicobacter*, *Pseudomonas*, *Bifidobacteria*, and *Enterobactereacae*. In contrast, these taxa were almost absent in the control group. Conversely, taxa such as *Mycobacteria*, *Neisseria*, *Rothia*, and *Actinomyces* showed more overrepresentation in the control group, however the adjusted False Discovery Rate (FDR) P- value showed no statistical significance indicating the need for further exploration (Fig. 4 and Supplementary Table 2).

#### Microbiota taxonomic analysis

**Fig. 5:**
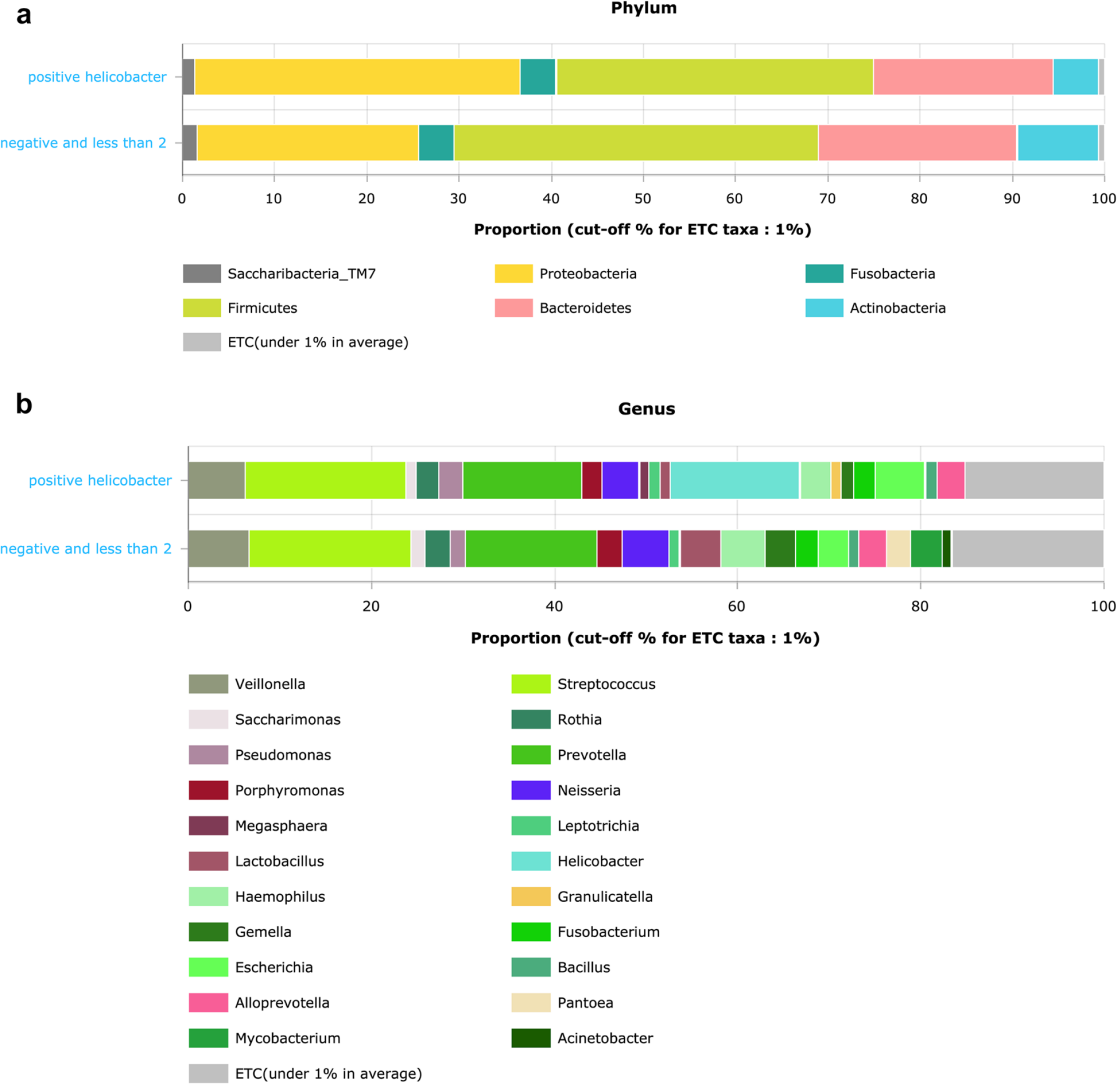
Distribution of microbial taxa among the *Helicobacter*-pos (RA ≥2) and -neg (RA<2) gastric biopsies. **a** average RA of microbial taxa at the phylum rank. **b** average RA of microbial taxa at the genus rank

The samples were classified into two groups: *H. pylori*-pos (RA ≥ 2%) and *H. pylori* -neg (RA < 2%), based on the RA of *H. pylori*. The RA of Proteobacteria and Firmicutes phyla significantly differed between both groups (*p* values = 0.002 and 0.021, respectively). In samples containing *H. pylori*, Proteobacteria predominated over Firmicutes; however, in samples lacking *H. pylori*, it ranked as the second most dominant phylum after Firmicutes (Fig. 5a). The *H. pylori*-pos samples showed lower F/B, compared to *H. pylori*-neg samples, with no significant difference (*p* value = 0.722). At the genus level, both groups showed the highest RA of *Streptococcus* and *Prevotella*, after which the third dominant genus was *Helicobacter* in the *H. pylori*-pos group, whereas *Veillonella* was dominant in the *H. pylori*-neg group (Fig. 5b). The LEfSe analysis of microbial taxa between both groups (Supplementary Fig. 5) indicated that *Helicobacter*, *Escherichia*, *Pseudomonas*, *Enterobacteriaceae*, and *Granulicatella* were the predominant taxa in the *H. pylori*-pos group. *Mycobacterium* and *Atopobium* genera exhibited the highest levels of abundance in the *H. pylori*-neg group.

#### Alpha and Beta diversity

**Fig. 6:**
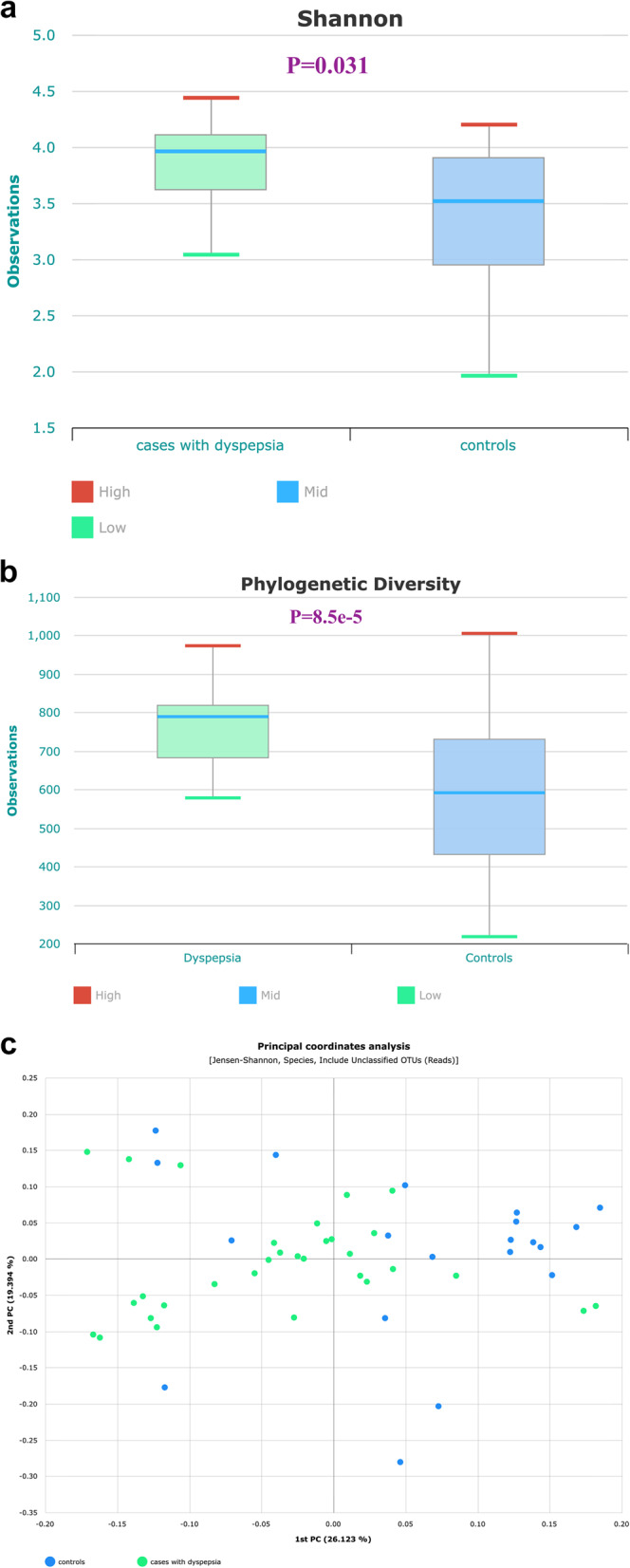
Bacterial diversities among gastric biopsies for cases of dyspepsia and controls. **a** Alpha diversity in dyspepsia and control groups represented by the Shannon index boxplot displaying the median and interquartile ranges with extended whiskers. **b** Microbial phylogenetic diversity boxplot in dyspepsia and control groups. **c** Beta diversity vsualized by publicationthe PCoA plot deduced by taxonomic composition profiles in gastric biopsies of dyspepsia and control groups. Each coordinate displays the percentage of diversity. The Beta diversity distance was measured by Bray-Curtis dissimilarity index

The paired antral and body gastric biopsies showed significantly higher bacterial diversity among dyspepsia cases than the controls. These results were obtained using the alpha diversity Shannon index (*p* value = 0.031), as well as the phylogenetic diversity index (*p* value = 8.5e-5), which involves the abundance and the phylogenetic tree distance (Fig. 6a and b). The *H. pylori* -pos and -neg samples revealed no significant difference in terms of alpha and the phylogenetic diversities (*p* value = 0.579) (Supplementary Fig. 6a and b).

In order to assess the resemblance of microbial composition among gastric biopsies of dyspepsia and control groups, the Bray-Curtis dissimilarity index, with plotted two-dimensional principal coordinate analysis (PCA) was used to estimate the Beta diversity based on the profile of microbiota RA. An obvious distinction was observed in the clustering of samples from the dyspepsia and control groups (Fig. 6c), as well as between *H. pylori*-pos and -neg groups (Supplementary Fig. 6c). This finding indicates a significant difference in the microbiota composition between these groups (*p* value = 0.001).

#### Functional analysis of gastric microbiota

A total of 153 functional pathways were identified using PICRUSt based on the KEGG core databases. Among these, 96 pathways showed significant differences in mean abundance between the dyspepsia and control groups as per the adjusted *p* value FDR (Supplementary Table 3). The majority of these relevant pathways belonged to functional categories of Metabolism (*n* = 60, predominantly Amino acid, Carbohydrate and Lipid metabolism), Genetic information and processing (*n* = 11, primarily Replication and repair), Organismal systems (*n* = 11, chiefly Immune and Endocrinal systems) and Human diseases (*n* = 9, mainly Cancer and Infectious bacterial diseases). Functional pathways significantly varying between dyspepsia and control groups are illustrated in Supplementary Fig. 7. The dyspepsia group revealed significant over enrichment of pathways such as: Epithelial cell signaling in *Helicobacter pylori* infection, Biosynthesis of nucleotide sugars, DNA replication, Ribosome, Mismatch repair, Pyrimidine metabolism, Peptidoglycan synthesis and Glycolysis/Gluconeogenesis (Supplementary Fig. 7).

In the current study, Firmicutes, Proteobacteria, and Bacteroidetes phyla showed the highest prevalence in dyspepsia and control groups, based on the microbial taxonomical analysis of gastric biopsies. Consistent with previous reports, these phyla are considered to be the most prominent microbiota in the gastrointestinal tract, accounting for > 98% of gut microbiota [3, 7, 18–21]. The distribution of these phyla was consistent in both the control and dyspepsia groups, but the RA of Proteobaceteria was significantly increased in the dyspepsia group. This finding aligns with previous researches indicating that Proteobacteria are commonly found in various gastric disorders, ranging from mild dyspepsia to gastric carcinomas. This suggests that the presence of Proteobacteria may indicate microbial dysbiosis in the gastrointestinal tract [22]. The increased prevalence of Proteobacteria in individuals with dyspepsia, as opposed to those without the condition, may be attributed to the overrepresentation of *Helicobacter* and *Prevotella* genera, which are part of this particular phylum, as reported by several studies [3, 23]. Nevertheless, a previous study observed the override of the Proteobacteria in *H. pylori*-pos and–neg cases [24]. In contrast, a previous study reported that Bacteroidetes had a higher RA that exceeded Proteobacteria in functional dyspepsia cases compared to the controls [10]. According to that study, functional dyspepsia may involve the movement of duodenal fluid back into the stomach, which can result in the transfer of Bacteroides (phylum: Bacteroidetes), which are known to be the main bacterial inhabitants of the intestine [10]. Nevertheless, additional verification is required for those findings due to the study’s focus on the gastric fluid, which contains microbial populations that are less stable and more vulnerable to the impact of bile acid, gastric acid, and the enzymes of the pancreas compared to the microbiota associated with the mucosa [10, 23]. Moreover, the gastric juice is subjected to either bacteria influxed from the oral cavity or refluxed through the duodenum [3].

Firmicutes and Bacteroidetes are regarded as the main phyla found in the gut microbiota. The F/B ratio can be changed in various pathological disorders as a result of an imbalance in the gut micro-ecology. This imbalance can increase or decrease the F/B ratio [25, 26]. The present study revealed a lower F/B ratio among cases of dyspepsia than controls. However, this difference was not statistically significant. This finding aligns with previous research indicating that dysbiosis is characterized by increased abundance of members of Bacteroidetes at the expense of those of the Firmicutes [10, 26].

At the genus rank, *Streptococcus*, *Prevotella*, and *Helicobacter* were of the highest abundance in both study groups. This aligns with numerous studies that reported *Streptococcus* and *Prevotella* as the top two predominant genera in gastric microbiota, despite the geographic, medical, and ethnical variances of the studied populations [17, 27–29]. Multiple studies have consistently identified *Streptococcus* and *Prevotella* as the primary types of bacteria associated with both normal and abnormal gastric disorders [17, 18, 21, 30]. Both genera typically exist among the oral cavity’s flora and the esophagus; it remains unclear whether their presence in the stomach is transient or permanent [21]. Few studies reported that cases with functional dyspepsia had a higher amount of *Streptococcus* bacteria and a lower amount of *Prevotella* bacteria. This result is attributed to the correlation between motor impairments and duodenal reflux of bile acids, which can hinder the growth of certain gastric bacteria such as *Prevotella* [31, 32].

*Helicobacter* was found to be the third most prevalent genus, following *Streptococcus* and *Prevotella*. The cases of dyspepsia showed significant overrepresentation of *Helicobacter* than the controls, which is in line with prior study that acknowledged the role played by *H. pylori* in the initiation and course of functional dyspepsia [33]. Patients with *H. pylori* infection are at an increased risk of developing functional dyspepsia [34]. However, eliminating the *H. pylori* infection can help alleviate the features of dyspepsia [35]. *H. pylori* has been incriminated in other gastric disorders, such as superficial gastritis, precancerous and cancerous lesions in the stomach [12, 36]. In addition, it stimulates the release of pro-inflammatory mediators that trigger an inflammatory response, which disturbs the stomach mucosal barrier and causes aberrations in the epithelial cells of the stomach, resulting in atrophic gastritis, gastric metaplasia, and carcinoma [7]. Sequencing can be used to detect *H. pylori*, but conventional methods like culture and urease tests may not always be successful in its detection [36].

However, finding sequences for *H. pylori* in gatric biopsies does not inevitably suggest an *H. pylori* infection. Therefore, it is crucial to establish a specific threshold value for *H. pylori* sequences, to accurately define an *H. pylori* infection [7, 36]. *H. pylori*-pos and -neg group specimens were assigned as those having *H. pylori* RA ≥ 2 and < 2, respectively [37]. Expectedly, we observed a significant higher RA of Proteobacteria among the *H. pylori* -pos group than the *H. pylori*-neg group, given that the *H. pylori* is the chief member in the Proteobacteria phylum. The Firmicutes exhibited an inverse pattern, potentially elucidating the lower F/B ratio observed in the *H. pylori*-pos group than the *H. pylori*-neg group [38]. The LEfSe analysis outlined that the *H. pylori*-pos group was primarily associated with *Helicobacter*, *Escherichia*,* Enterobacteriaceae*, *Granulicatella*, and *Staphylococcus* genera. Unlikely, other genera, such as *Mycobacterium* and *Atopobium* showed a decrease in the RA among the *H. pylori*-pos group. This finding was supported by previous studies that examined the variation in microbial taxa between *H. pylori*-pos and -neg groups [38, 39].

Previously, it has been hypothesized that *H. pylori* does not sufficiently impact stomach microbiota [28]. Recent studies have contradicted this by discovering notable changes in the stomach microbiota of *H. pylori*-pos individuals, providing an increasing evidence for the alteration of stomach microbiota under the influence of *H. pylori* [27, 40]. One possible explanation is that *H. pylori* may alter the pH and damage the mucosa of the stomach, which results in forming niches that allow the overgrowth of other types of bacteria. That disturbs the symbiosis in the stomach [11, 18]. Moreover, certain studies have established a relation between disturbance of microbiota and the impact of *H. pylori* on immune responses, as evidenced by the increase of several inflammatory mediators and T-cell responses, which ultimately disrupt existing microbiota [41].

Regarding the specific gut taxa, our study revealed that *E. coli*, *Pseudomonas*, *Bifidobacterium*, and Enterobacteriaceae were the highest discriminatory taxa for dyspepsia compared to controls as proven by the LEfSe analysis. Furthermore, the presence of *Clostridium* and *Bacteroides* was significantly higher in cases of dyspepsia compared to controls despite their low abundance. Our results were consistent with prior researches that found an abundance of these microbial taxa in gastric mucosa of cases of dyspepsia [10, 11, 23, 32]. These microbial taxa, which are commonly found in the intestine, are considered abnormal when they are present in higher abundance in the stomach, particularly in cases of dyspepsia. The occurrence of dyspepsia can be attributed to small intestinal bacterial overgrowth (SIBO), which is caused by disrupted gastrointestinal motility. This disruption leads to retrograde movement of bacteria of the intestinal type to the stomach [19]. From a different perspective, changes in the microbiota can impact gastric motility. They may contribute to the underlying pathophysiology of functional dyspepsia, where the toxic metabolites and cellular components of migrated bacteria stimulate the gastric secretion of inflammatory cytokines, enhancing mucosal permeability and disturbing the gastric nerves [7, 10, 12, 23]. Our study revealed a higher abundance of *Lactobacillus mucosae* among patients with dyspepsia, which is consistent with findings from other studies [11, 42]. Some *Lactobacillus* species have probiotic properties that restore the balance in the gastric microbiome [7, 12]. Nevertheless, several studies revealed an increased overall RA of *Lactobacillus* in gastritis and along the stages of gastric carcinoma [29, 36].

Across literature, a decrease in microbial alpha diversity has consistently been identified as an indicator of microbial dysbiosis. This decrease is associated with a higher likelihood of developing gastric disorders [21]. However, our study revealed a discrepancy as we observed higher alpha diversity among cases of dyspepsia than controls. This is concordant with several studies that have either reported absent difference in alpha diversity between both groups or higher diversity among the cases of dyspepsia [31, 43]. A prior study found that cases of dyspepsia had a significantly higher alpha diversity was found among cases of dyspepsia than controls at the species level. However, no significant difference was demonstrated at the genus level indicating that microbial expansion may occur at one taxonomic level without affecting the other [43]. Other influencing factors may elucidate the contradictory findings of alpha diversity, such as the type of the study population, level of modernization, and dietary habits [44, 45]. No remarkable difference in alpha diversity was shown between the *H. pylori*-pos and *H. pylori*-neg groups. This finding agrees with one study showing that *H. pylori* did not negatively impact the diversity of the gastric microbial community [28]. In contrast, other studies observed reduced alpha diversity among *H. pylori*-pos cases [22]. The disparities among studies could be attributed to factors like duration of *H. pylori* infection, active virulence factors, host immune response, and dietary habits [46]. Our study found significant dissimilarity in microbial structure between the groups of dyspepsia and controls groups, and likely between *H. pylori*-pos and -neg groups. This supports previous reports that have identified the presence of dysbiosis in gastric disorders caused by changes in the ecological conditions of the stomach [10, 27, 31, 32, 38, 39]. The functional analysis of mucosa-associated gastric microbiota indicated overall predominance of pathways related to metabolism, genetic information processing, organismal systems and infectious diseases. The dyspepsia group exhibited substantial override of pathways including Epithelial signaling in *H. pylori*, DNA replication, Peptidoglycan synthesis and Glycolysis/Gluconeogenesis compared to controls. However, very limited data is available in literature on the functional background of dyspepsia-associated microbiota, with the majority of data focusing on the entire gastrointestinal tract rather than the gastric mucosa [18].

## Discussion

Previous studies have demonstrated the profile of abundant microbiota in the stomach. However, the intricate nature of the microbiome, its continuous evolution, and the undiscovered functional host/inter-microbial pathophysiological mechanisms make it challenging to reach a consensus about its role in health and disease [7, 23]. The discrepancies among studies can be ascribed to variations in i) study populations with divergences in geographical, ethnic, and cultural characteristics, ii) types of samples, including gastric fluid, gastric biopsies, or stool, and iii) the laboratory technique utilized to examine the microbiota. Prior studies were primarily addressing the gastrointestinal microbiota present in less- specific gastric fluid or fecal matter, where microbial populations are less stable and more vulnerable to various influencing factors such as bile acid, gastric acid and stomach enzymes [10, 23, 47]. Our study present an added significant value of investigating altered mucosa- associated microbiome in dyspepsia cases by utilizing site-specific gastric tissue specimens (antrum and body) enabling comprehensive and robust profiling of complex microbial communities. However, our study was limited by low sample size due to the invasive nature of sample collection. Hence, the study is regarded as a pilot study for primary exploration of relevant shifts in mucosa-associated gastric in a limited cohort of functional dyspepsia cases. This implies interpreting all statistical findings with caution and indicates the need for further expanded large-scale studies to draw evidence-based conclusions. Despite the merits of 16 srRNA gene sequencing, it does not distinguish between live and dead organisms, nor address gene expression, resulting products and their interactions within metabolic pathways. Future research can employ advanced technologies like shot-gun sequencing, functional metabolomics and transcriptomics to gain enhanced comprehension of microbiome functionality in the stomach and develop more specific mechanism-based tailored therapies.

## Conclusion

Firmicutes, Proteobacteria, and Bacteroides phyla, as well as *Streptococcus*, *Prevotella*, and *Helicobacter* genera were the most abundant microbial taxa among all gastric biopsies of both dyspepsia and control groups. The phylum Proteobacteria and its primary genus, *Helicobacter*, were found in higher proportions in the biopsies of the dyspepsia group. The species of *H. pylori* and *Prevotella melaninogenica* were significantly more abundant in the dyspepsia compared to the control group. The dyspepsia group showed high discrimination for several vital taxa, including *E. coli*, *Pseudomonas*, *Bifidobacteria*, and *Enterobacteriaceae*, in addition to a dissimilarity in microbial composition, compared to the control group. Future studies should address functional metagenomics to enhance our comprehension of the microbial ecology in the gastrointestinal tract, leading to more mechanism-based tailored therapies.

## Supplementary Information

Supplementary Material 1.

Supplementary Material 2.

## Data Availability

The datasets generated and/or analysed during the current study are available in the NCBI, under Bioproject number: PRJNA122653, with the detailed accession number for datasets in supplementary file 2.

## Abbreviations

NGS: Next-generation sequencing
OUT: Operation taxonomic units
PCoA: Principal Coordinate Analysis
LEfSe: Linear discriminant analysis effect size
FDR: False Discovery Rate
KEGG: Kyoto Encyclopedia of Genes and Genomes
PICRUSt: Phylogenetic investigation of communities by reconstruction of unobserved states
