# Supplementary material for: Analysis of gastric mucosa associated microbiota in functional dyspepsia using 16S rRNA gene next-generation sequencing
Source: BMC Microbiol. 2025 Jun 26;25:368. doi: 10.1186/s12866-025-04095-0 (PMC12199507; doi:10.1186/s12866-025-04095-0)
Supplement: Supplementary file 1 — Supplementary Material 1. [file 12866_2025_4095_MOESM1_ESM.pdf]

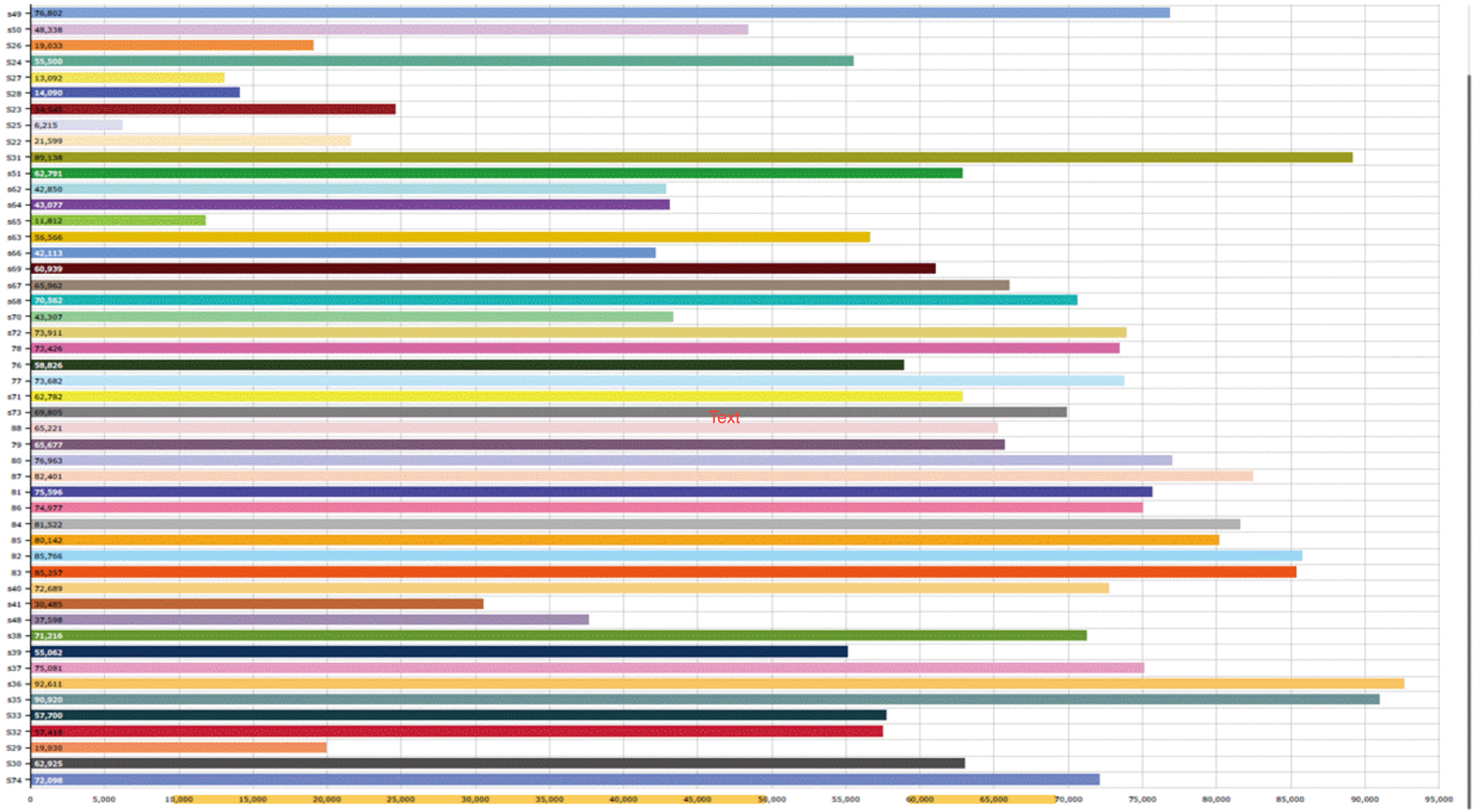

Supplementary Figure 1: The number of the valid reads each of the total tested samples (n=30)

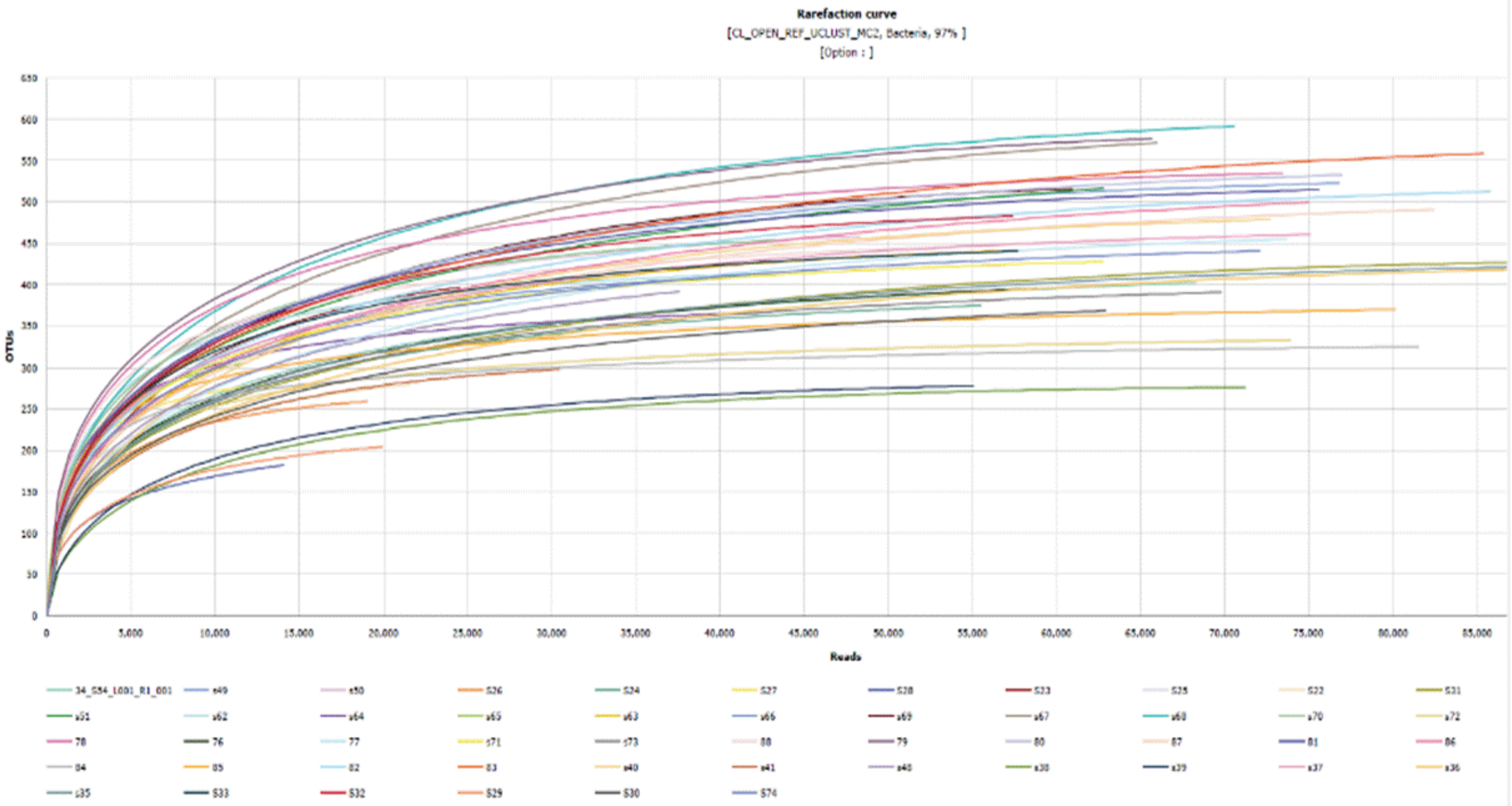

Supplementary Figure 2: Rarefaction curves of valid reads for OTUs. Each line with a certain color represents one sample

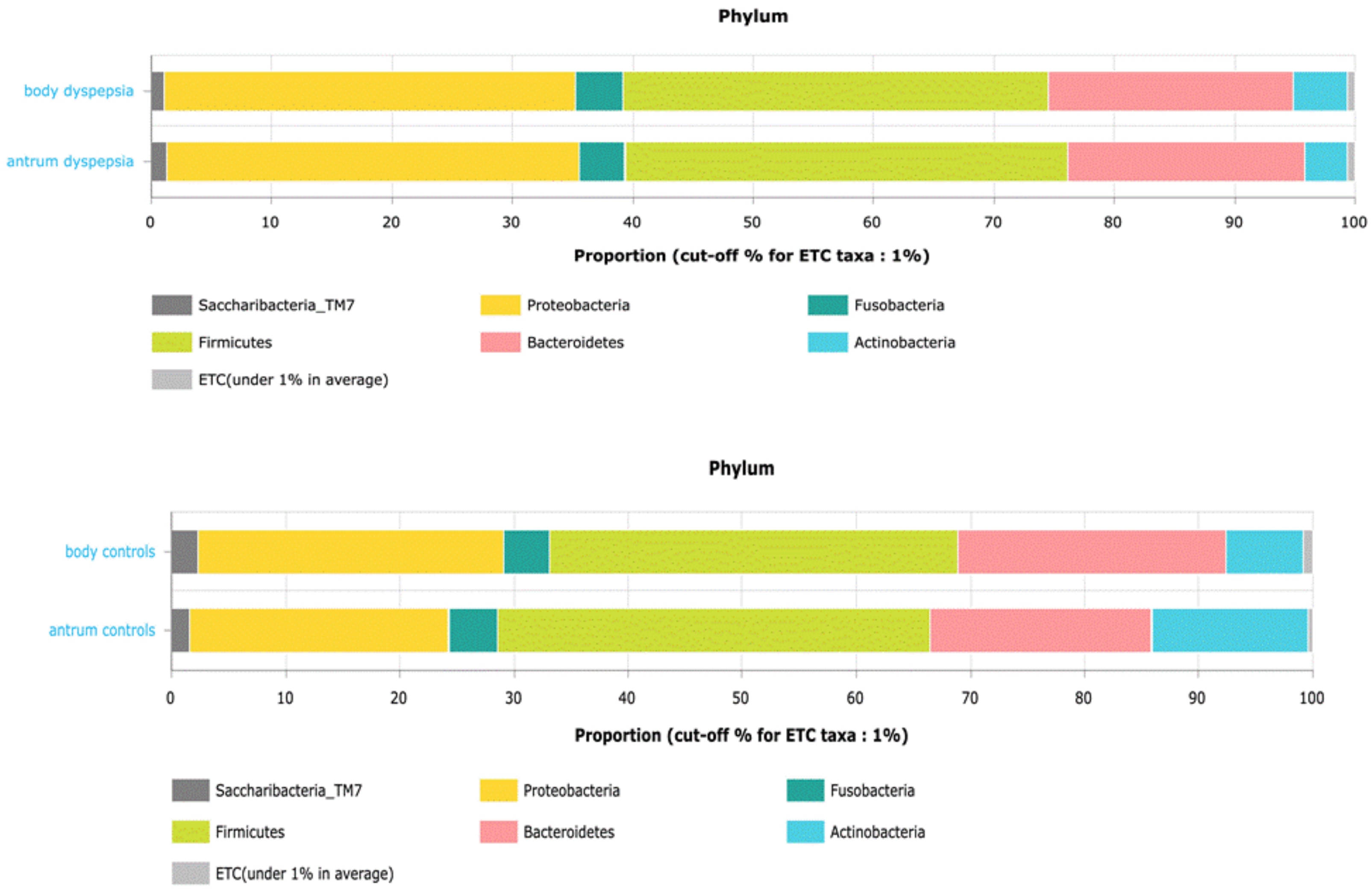

Supplementary Figure 3: Distribution of microbial phyla according to the average RA among antral and body biopsies of both dyspepsia and control groups

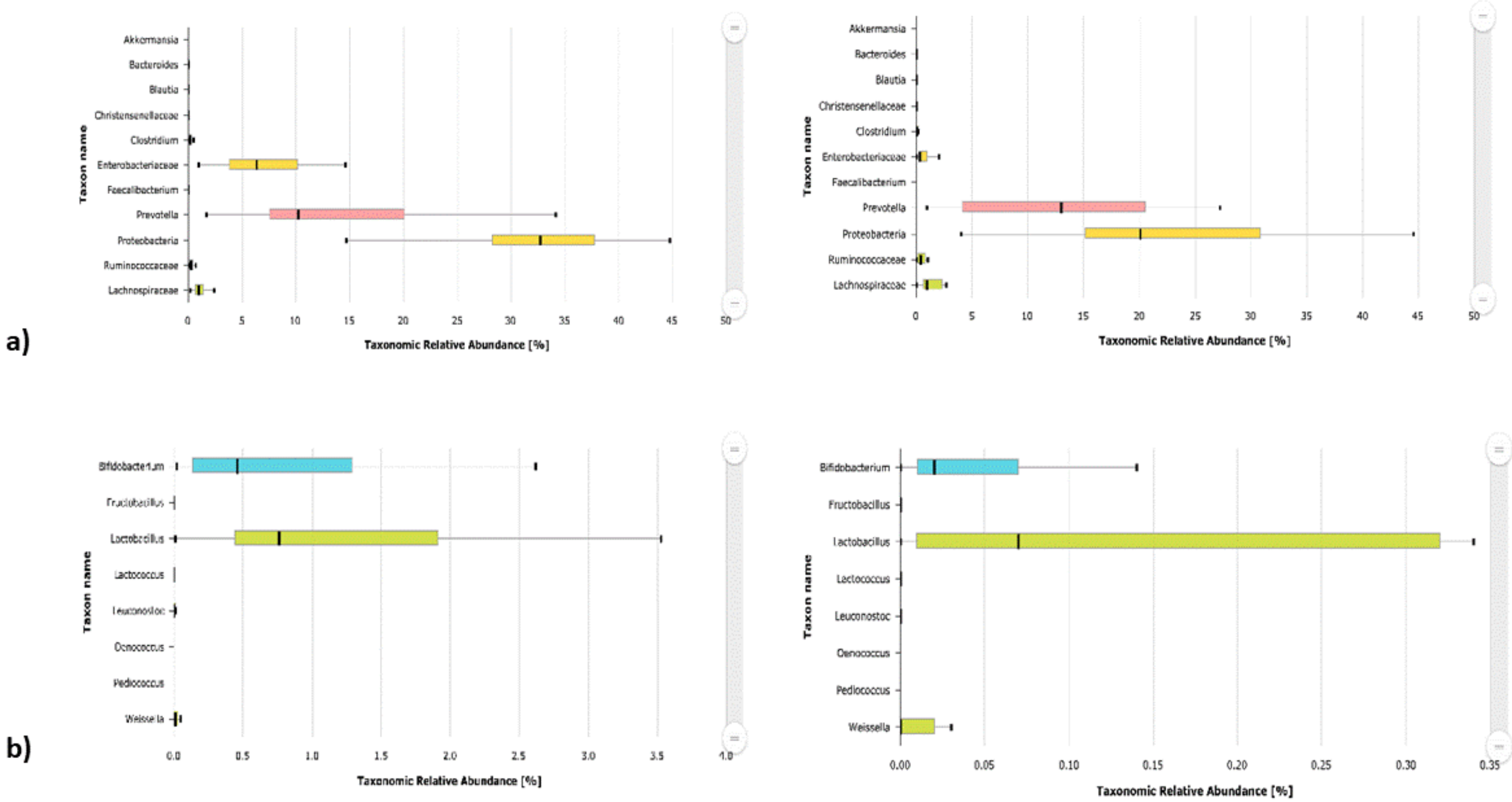

Supplementary Figure 4: Distribution of human gut taxa among gastric biopsies of dyspepsia and control groups. a) Average RA of human gut taxa among gastric biopsies of dyspepsia (left) and control groups. b) Lactic acid bacteria composition among gastric biopsies of dyspepsia cases (left) and controls (Right)

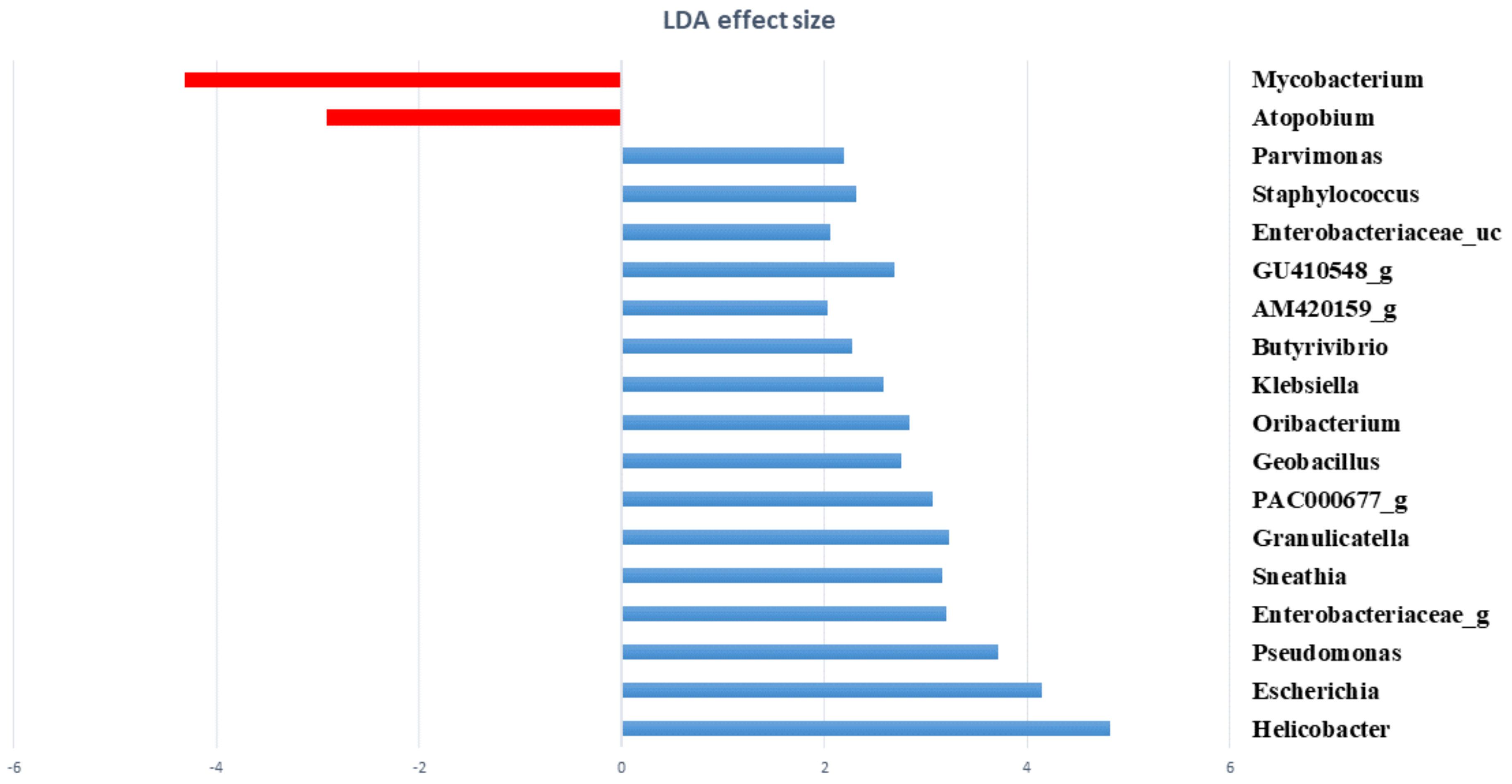

Supplementary Figure 5: The highest relevant discriminatory microbial taxa between the between H. pylori-pos and H. pylori-neg gastric biopsies. Linear discriminative analysis (LDA) effect size cladogram showing relevant taxa with the highest discrimination between H. pylori-pos and H. pylori-neg gastric biopsies. The microbial taxa are sorted in descending order of their log 10 LDA score.

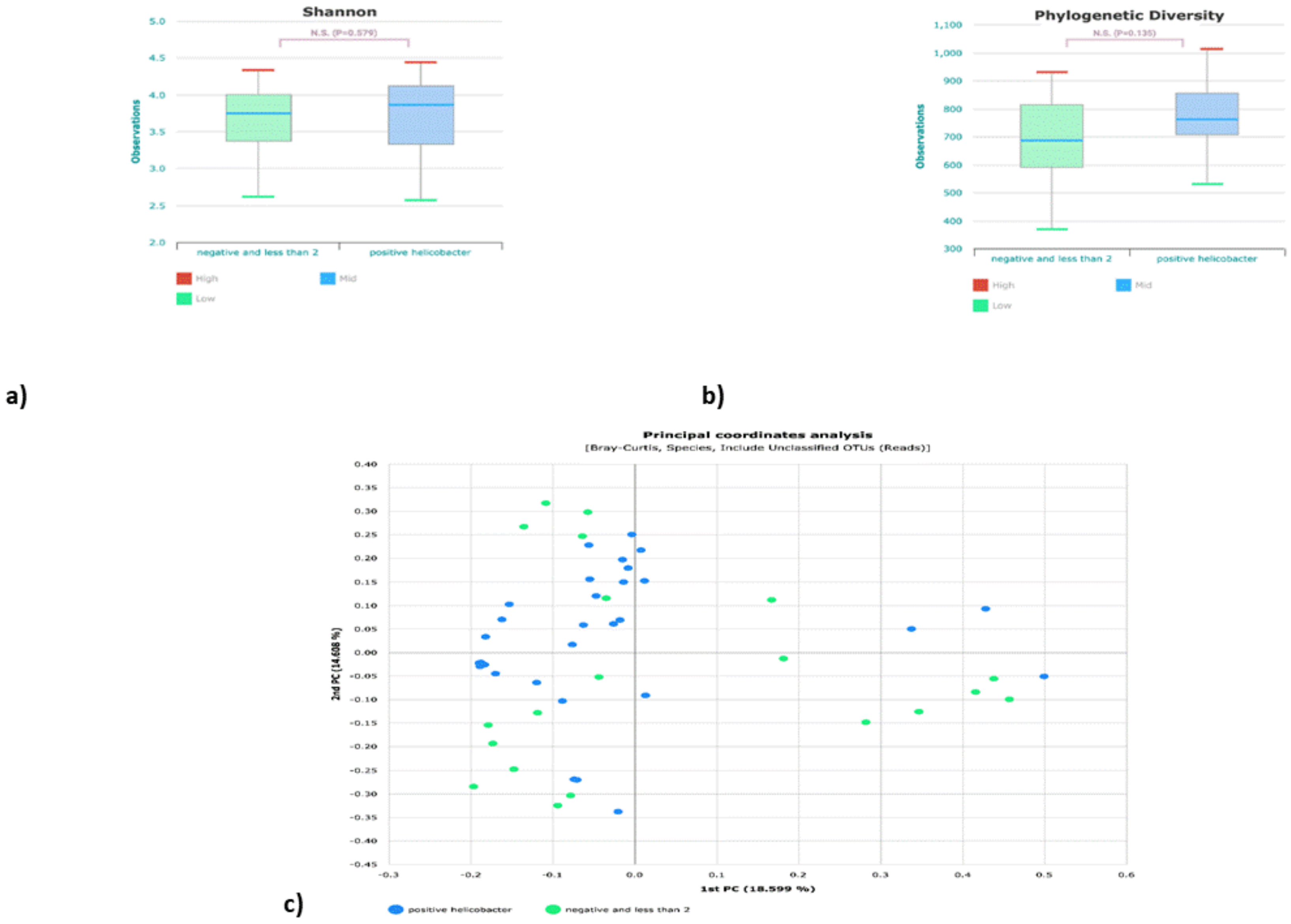

Supplementary Figure 6: : Bacterial diversities among gastric biopsies for H. pylori-pos and H. pylori-neg gastric biopsies. a) Shannon index boxplot for the microbial alpha diversity in H. pylori-pos and H. pylori-neg gastric biopsies, b) Microbial phylogenetic diversity boxplot in H. pylori-pos and H. pylori-neg groups, c) Microbial Beta diversity PCOA plot based on taxonomic composition profile using Bray-Curtis measure based on taxonomic composition profile of H. pylori-pos and H. pylori-neg gastric biopsies. Each coordinate displays the % of diversity

Supplementary Table 1: Number of valid reads among tested samples

MTP_List

MTP ID	MTP name	Region	Valid reads	DB Ver.
FR23013000S0021	S25	V3V4	6,215	PKSSU4.0
FR23012900S0036	s65	V3V4	11,812	PKSSU4.0
FR23013000S0019	S27	V3V4	13,092	PKSSU4.0
FR23013000S0018	S28	V3V4	14,090	PKSSU4.0
FR23013000S0020	S26	V3V4	19,033	PKSSU4.0
FR23013000S0017	S29	V3V4	19,930	PKSSU4.0
FR23013000S0024	S22	V3V4	21,599	PKSSU4.0
FR23013000S0023	S23	V3V4	24,545	PKSSU4.0
FR23013000S0003	s41	V3V4	30,485	PKSSU4.0
FR23013000S0002	s48	V3V4	37,598	PKSSU4.0
FR23012900S0035	s66	V3V4	42,113	PKSSU4.0
FR23012900S0039	s62	V3V4	42,850	PKSSU4.0
FR23012900S0037	s64	V3V4	43,077	PKSSU4.0
FR23012900S0031	s70	V3V4	43,307	PKSSU4.0
FR23012900S0041	s50	V3V4	48,338	PKSSU4.0
FR23013000S0005	s39	V3V4	55,062	PKSSU4.0
FR23013000S0022	S24	V3V4	55,500	PKSSU4.0
FR23012900S0038	s63	V3V4	56,566	PKSSU4.0
FR23013000S0011	S32	V3V4	57,418	PKSSU4.0
FR23013000S0010	S33	V3V4	57,700	PKSSU4.0
FR23012900S0026	76	V3V4	58,826	PKSSU4.0
FR23012900S0032	s69	V3V4	60,939	PKSSU4.0
FR23012900S0029	s71	V3V4	62,782	PKSSU4.0
FR23012900S0040	s51	V3V4	62,791	PKSSU4.0
FR23013000S0016	S30	V3V4	62,925	PKSSU4.0
FR23012900S0024	79	V3V4	65,677	PKSSU4.0
FR23012900S0034	s67	V3V4	65,962	PKSSU4.0
FR23013100S0176	34_S54_L001_R1_001	V3V4	68,280	PKSSU4.0
FR23012900S0028	s73	V3V4	69,805	PKSSU4.0
FR23011900S0015	S75	V3V4	70,181	PKSSU4.0
FR23012900S0033	s68	V3V4	70,562	PKSSU4.0
FR23013000S0006	s38	V3V4	71,216	PKSSU4.0
FR23011900S0016	S74	V3V4	72,098	PKSSU4.0
FR23013000S0004	s40	V3V4	72,689	PKSSU4.0
FR23012900S0025	78	V3V4	73,426	PKSSU4.0
FR23012900S0027	77	V3V4	73,682	PKSSU4.0
FR23012900S0030	s72	V3V4	73,911	PKSSU4.0
FR23012900S0017	86	V3V4	74,977	PKSSU4.0
FR23013000S0007	s37	V3V4	75,081	PKSSU4.0
FR23012900S0022	81	V3V4	75,596	PKSSU4.0
FR23012900S0042	s49	V3V4	76,802	PKSSU4.0
FR23012900S0023	80	V3V4	76,963	PKSSU4.0
FR23012900S0018	85	V3V4	80,142	PKSSU4.0
FR23012900S0019	84	V3V4	81,522	PKSSU4.0
FR23012900S0016	87	V3V4	82,401	PKSSU4.0

Supplementary Table 2: Linear discriminate analysis effect size (LEfSe) analysis of microbial composition among dyspepsia and control groups

Taxon name	Taxon	LDA eff	p-value	p-(FDR)	dysp.	cont.
Proteobacteria	Phylum	4.71470	0.00384	0.09470	34.17000	24.43500
Gammaproteobacteria	Class	4.65484	4.335e-6	0.00062	18.12667	9.20000
Enterobacteriaceae	Family	4.57728	3.632e-8	0.00002	8.58333	0.72500
Actinobacteria_c	Class	4.55352	0.03666	0.37050	$3.71	10.02000
Escherichia	Genus	4.51909	1.798e-8	0.00001	7.24000	0.36500
Escherichia coli group	Species	4.51882	1.798e-8	0.00001	7.23000	0.36500
Enterobacterales	Order	4.45146	8.947e-7	0.00028	8.72000	3.41000
Mycobacterium	Genus	4.38799	0.00448	0.09747	0.11333	3.41500
Mycobacterium farcinog	Species	4.37757	0.00448	0.09747	0.11333	3.41500
Corynebacteriales	Order	4.37075	0.00243	0.06702	0.27667	3.47000
Campylobacterales	Order	4.35716	0.01794	0.23351	10.65333	7.57000
Epsilonproteobacteria	Class	4.35716	0.01794	0.23351	10.65333	7.57000
Helicobacteraceae	Family	4.34249	0.03289	0.35051	9.79333	7.11500
Helicobacter	Genus	4.34246	0.03289	0.35051	9.79000	7.11500
Helicobacter pylori grou	Species	4.34226	0.03289	0.35051	9.78667	7.11500
Mycobacteriaceae	Family	4.33267	0.00448	0.09747	0.11333	3.41500
Neisseria	Genus	4.19620	0.03574	0.37050	3.46000	6.38000
Neisseriaceae	Family	4.19312	0.03752	0.37050	3.55667	6.46000
Neisseriales	Order	4.19312	0.03752	0.37050	3.55667	6.46000
Rothia	Genus	4.18626	0.01151	0.17797	1.26667	4.49000
Micrococcaceae	Family	4.18430	0.00769	0.13264	1.34333	4.55000
Micrococcales	Order	4.17196	0.00791	0.13492	1.49333	4.61000
Betaproteobacteria	Class	4.15951	0.04135	0.38952	4.36333	7.00000
KV831974_s group	Species	4.06792	0.01529	0.21540	0.53667	2.61500
Pseudomonadales	Order	4.02968	0.00008	0.00497	3.20000	0.98000
Pseudomonas	Genus	4.00507	0.00011	0.00651	2.87000	0.78500
Pseudomonadaceae	Family	4.00010	0.00011	0.00651	2.87000	0.78500
Lactobacillaceae	Family	3.98954	0.00097	0.03484	2.52333	2.71500
Lactobacillus	Genus	3.96856	0.00097	0.03484	2.52333	2.71500
Lactobacillus mucosae	Species	3.90979	2.999e-6	0.00048	1.66333	0.02500
Pseudomonas stutzeri g	Species	3.89693	0.00024	0.01248	2.38333	0.75000
Actinomycetaceae	Family	3.80714	0.00373	0.09383	0.51667	1.78000
Actinomycetales	Order	3.80571	0.00373	0.09383	0.51667	1.78000
Actinomyces	Genus	3.80157	0.00362	0.09383	0.50667	1.76000
Bifidobacterium	Genus	3.74048	4.842e-6	0.00063	1.17000	0.05500
Bifidobacteriaceae	Family	3.72995	0.00002	0.00180	1.17667	0.09000
Bifidobacteriales	Order	3.72961	0.00002	0.00180	1.17667	0.09000
Bacillaceae	Family	3.68843	0.00940	0.15659	2.17333	1.16000
Veillonella rogosae	Species	3.67342	0.00152	0.05304	0.44000	1.32000
Bifidobacterium longum	Species	3.60965	1.070e-6	0.00028	0.83667	0.01500
Rothia mucilaginosa gro	Species	3.60252	0.00367	0.09383	0.56667	1.44500
Haemophilus influenzae	Species	3.59511	1.552e-6	0.00028	1.01000	0.25000
Xanthomonadaceae	Family	3.57623	0.00197	0.06106	1.27667	0.49500
Xanthomonadales	Order	3.56912	0.00197	0.06106	1.27667	0.49500
Enterobacteriaceae grou	Species	3.55988	0.00002	0.00170	0.99667	0.23000

Enterobacteriaceae_g	Genus	3.55981	0.00002	0.00170	0.99667	0.23000
Bacillus	Genus	3.50999	0.00855	0.14397	1.41667	0.71500
Prevotella salivae	Species	3.49173	0.03716	0.37050	1.42333	0.78000
AY920317_s	Species	3.47487	0.01240	0.18693	1.26000	0.61000
Prevotella jejuni	Species	3.43772	0.01781	0.23351	1.61000	1.39000
Veillonella parvula grou	Species	3.39537	0.01219	0.18563	0.36333	0.88500
FJ976422_s	Species	3.36446	0.01624	0.21939	0.09000	0.49500
Fusobacterium nucleatu	Species	3.25012	0.03055	0.34446	0.92000	0.63500
Porphyromonas endodo	Species	3.20014	0.03656	0.37050	0.45000	0.12500
Staphylococcaceae	Family	3.19156	0.00063	0.02811	0.41667	0.09000
Hydrogenophilaceae	Family	3.17522	0.04061	0.38513	0.82333	0.52000
Hydrogenophilales	Order	3.16548	0.03964	0.37839	0.82667	0.52000
Hydrogenophilalia	Class	3.15797	0.03964	0.37839	0.82667	0.52000
Megasphaera elsdenii	Species	3.13274	0.00005	0.00339	0.29333	0.00500
Schlegelella aquatica	Species	3.12546	0.01642	0.21971	0.55667	0.28000
Vulcaniibacterium	Genus	3.11515	0.01343	0.19228	0.48000	0.20500
Schlegelella	Genus	3.11288	0.01556	0.21639	0.56000	0.28000
Pseudomonas fulva grou	Species	3.10988	1.422e-6	0.00028	0.27667	0.00000
Actinomyces odontolyti	Species	3.10947	1.437e-6	0.00028	0.01667	0.29500
Prevotella aurantiaca	Species	3.09661	0.00221	0.06196	0.08000	0.36500
Vulcaniibacterium therm	Species	3.09591	0.01343	0.19228	0.48000	0.20500
Enterococcaceae	Family	3.07386	0.00005	0.00339	0.25000	0.02500
Enterococcus	Genus	3.07107	0.00005	0.00339	0.25000	0.02500
Rothia dentocariosa	Species	3.06520	0.00214	0.06135	0.13000	0.36500
PAC001350_s	Species	3.06040	0.01292	0.18900	0.46667	0.31000
Enterococcus faecium gr	Species	3.04618	0.00007	0.00430	0.23333	0.02000
Bifidobacterium breve	Species	3.04233	0.00013	0.00704	0.21667	0.00000
Stenotrophomonas	Genus	3.00341	0.00029	0.01408	0.25333	0.05500
FWNZ_s	Species	2.99072	0.00209	0.06106	0.27000	0.07000
Staphylococcus	Genus	2.98576	0.00087	0.03458	0.27333	0.08500
Megasphaera indica	Species	2.97596	0.00006	0.00428	0.18667	0.00000
Tepidiphilus	Genus	2.97375	0.04442	0.41309	0.57667	0.35500
Klebsiella	Genus	2.96945	0.00209	0.06106	0.27000	0.07000
DQ087194_s	Species	2.96935	0.03224	0.35051	0.09000	0.31000
Capnocytophaga leadbe	Species	2.96563	0.00454	0.09747	0.27000	0.07000
Staphylococcus aureus g	Species	2.95432	0.00082	0.03359	0.26333	0.08000
Stenotrophomonas malt	Species	2.95319	0.00039	0.01878	0.23000	0.05500
Peptostreptococcus	Genus	2.95259	0.00003	0.00283	0.24667	0.06000
Peptostreptococcus stor	Species	2.93383	0.00003	0.00283	0.24333	0.06000
Cutibacterium	Genus	2.91198	0.00022	0.01161	0.22333	0.05000
Propionibacteriales	Order	2.90158	0.00056	0.02609	0.23667	0.06500
Acinetobacter	Genus	2.90130	0.00419	0.09747	0.29667	0.12500
Sneathia	Genus	2.89001	0.00576	0.10994	0.26667	0.14000
AJ289183_s	Species	2.88114	0.00539	0.10871	0.26000	0.13500
Cutibacterium acnes grc	Species	2.88007	0.00091	0.03484	0.21000	0.05000
Propionibacteriaceae	Family	2.87924	0.00082	0.03359	0.22667	0.06500
Sphingobacteriales	Order	2.84449	0.03644	0.37050	0.25333	0.12000

Peptostreptococcaceae	Family	2.83630	0.00448	0.09747	0.27000	0.12500
Sphingobacteriia	Class	2.83540	0.03789	0.37159	0.26000	0.12500
Spirochaetes	Phylum	2.83402	0.00727	0.13006	0.27333	0.26500
Erysipelotrichi	Class	2.81879	0.00194	0.06106	0.23000	0.10500
Erysipelotrichaceae	Family	2.81629	0.00194	0.06106	0.23000	0.10500
Treponema	Genus	2.80993	0.00612	0.11535	0.26333	0.26000
Spirochaetales	Order	2.80647	0.00727	0.13006	0.27333	0.26500
Erysipelotrichales	Order	2.80197	0.00194	0.06106	0.23000	0.10500
Spirochaetia	Class	2.79772	0.00727	0.13006	0.27333	0.26500
Acinetobacter baumann	Species	2.79757	0.00201	0.06106	0.19333	0.06500
Spirochaetaceae	Family	2.79617	0.00727	0.13006	0.27333	0.26500
Bulleidia	Genus	2.76688	0.00510	0.10430	0.21333	0.10500
Solobacterium moorei	Species	2.75646	0.00510	0.10430	0.21333	0.10500
Corynebacteriaceae	Family	2.75510	0.02977	0.34446	0.15333	0.04000
Oribacterium asaccharo	Species	2.74313	0.01900	0.24513	0.19333	0.07500
Streptococcus gordonii g	Species	2.73968	0.00070	0.03050	0.13000	0.03000
Corynebacterium	Genus	2.73859	0.02977	0.34446	0.15333	0.04000
Peptoniphilaceae	Family	2.73794	0.00569	0.10994	0.16000	0.04500
Prevotella scopos	Species	2.73740	0.02348	0.28985	0.01333	0.12500
Tissierellia	Class	2.73688	0.00569	0.10994	0.16000	0.04500
CP016753_s	Species	2.73662	0.01156	0.17797	0.00000	0.11500
Tissierellales	Order	2.73334	0.00569	0.10994	0.16000	0.04500
Lactobacillus reuteri gro	Species	2.72092	0.03041	0.34446	0.00000	0.12500
AM930290_g	Genus	2.70401	0.03304	0.35051	0.20333	0.11500
Chromatiales	Order	2.69951	0.00960	0.15794	0.14667	0.06000
PAC001344_s	Species	2.69737	0.04873	0.44449	0.14667	0.07500
Pseudomonas aerugino	Species	2.69017	0.00094	0.03484	0.09333	0.00500
AM930290_s	Species	2.68441	0.03304	0.35051	0.20333	0.11500
Thermomonas_uc	Species	2.66215	0.03224	0.35051	0.21000	0.11000
Chitinophagaceae	Family	2.65196	0.04217	0.39466	0.20667	0.12000
Thermomonas	Genus	2.64629	0.03224	0.35051	0.21000	0.11000
Bifidobacterium catenul	Species	2.63999	0.00456	0.09747	0.08333	0.00000
Streptococcus sanguinis	Species	2.60577	0.04829	0.44332	0.11667	0.05000
AZHT_s	Species	2.58166	0.00463	0.09747	0.09000	0.01500
Parvimonas micra	Species	2.53603	0.00256	0.06910	0.06667	0.00000
AM420159_g	Genus	2.51938	0.02721	0.33300	0.06667	0.02000
JN713551_s	Species	2.51404	0.02150	0.26777	0.05000	0.00000
Tepidiphilus_uc	Species	2.51085	0.01534	0.21540	0.08000	0.03000
Streptobacillus_uc	Species	2.51068	0.03038	0.34446	0.00000	0.03500
Enterobacteriaceae_uc	Genus	2.50761	0.01034	0.16821	0.05667	0.00500
Yersiniaceae	Family	2.46831	0.00448	0.09747	0.05000	0.00000
Serratia	Genus	2.46046	0.00448	0.09747	0.05000	0.00000
Streptococcus agalactiae	Species	2.44618	0.00767	0.13264	0.04000	0.00000
Mollicutes	Class	2.43060	0.01594	0.21736	0.07333	0.02000
Tenericutes	Phylum	2.41817	0.01594	0.21736	0.07333	0.02000
JQ450951_s	Species	2.40782	0.03031	0.34446	0.00000	0.01500
Atopobium rimae group	Species	2.40558	0.01147	0.17797	0.00000	0.02000

Anaeroglobus geminatu	Species	2.36940	0.01147	0.17797	0.00000	0.02000
Pseudomonas veronii gr	Species	2.35859	0.01293	0.18900	0.03667	0.00000
Aerococcus	Genus	2.35522	0.03934	0.37839	0.05333	0.01000
Enterobacterales_uc	Family	2.35378	0.02129	0.26741	0.02667	0.00000
AY349371_s	Species	2.35334	0.03031	0.34446	0.00000	0.01500
Bifidobacterium dentiur	Species	2.34999	0.01155	0.17797	0.00000	0.04000
Prevotella bivia	Species	2.34912	0.00761	0.13264	0.03667	0.00000
Serratia liquefaciens gro	Species	2.34857	0.01276	0.18900	0.03333	0.00000
Corynebacterium kroppe	Species	2.34763	0.04735	0.43741	0.04333	0.00500
Aerococcus viridans grou	Species	2.34652	0.03934	0.37839	0.05333	0.01000
Bifidobacterium_uc	Species	2.34151	0.02129	0.26741	0.02667	0.00000
Thermoanaerobacterale	Order	2.33574	0.03031	0.34446	0.00000	0.01500
Corynebacterium tuberc	Species	2.33226	0.02111	0.26741	0.02333	0.00000
Massilia	Genus	2.30524	0.03038	0.34446	0.00000	0.02000
Sporomusaceae	Family	2.24642	0.03031	0.34446	0.00000	0.01500
Rhizobiaceae	Family	2.12752	0.03701	0.37050	0.01667	0.03000
Rhizobium	Genus	2.11670	0.03701	0.37050	0.01667	0.03000
Rhizobium leguminosaru	Species	2.10952	0.03701	0.37050	0.01667	0.03000

Supplementary Table 3: Annotated functional pathways across dyspepsia and control groups

Pathway	Definition	p-value	p- (FDR)	Dysp.	Cont.
ko05120	Epithelial cell signaling in Helicobacter pylori infection	4E-05	0.0165	0.2079	0.0754
ko00970	Aminoacyl-tRNA biosynthesis	0.0009	0.0221	1.0575	0.8631
ko01250	Biosynthesis of nucleotide sugars	0.0008	0.0221	1.1157	0.9682
ko03018	RNA degradation	0.0009	0.0221	0.5238	0.4427
ko00541	O-Antigen nucleotide sugar biosynthesis	0.0009	0.0221	0.4916	0.4339
ko00740	Riboflavin metabolism	0.0005	0.0221	0.2959	0.2525
ko05206	MicroRNAs in cancer	0.0003	0.0221	0.0769	0.0499
ko05132	Salmonella infection	0.0009	0.0221	0.1678	0.1415
ko00760	Nicotinate and nicotinamide metabolism	0.0002	0.0221	0.4581	0.4367
ko00908	Zeatin biosynthesis	0.0007	0.0221	0.0515	0.038
ko04210	Apoptosis	0.0005	0.0221	0.0274	0.0169
ko05205	Proteoglycans in cancer	0.0009	0.0221	0.0431	0.0346
ko05340	Primary immunodeficiency	0.0002	0.0221	0.0446	0.038
ko00220	Arginine biosynthesis	0.0006	0.0221	0.3448	0.3907
ko01210	2-Oxocarboxylic acid metabolism	0.0005	0.0221	0.5345	0.5881
ko00340	Histidine metabolism	0.0006	0.0221	0.2079	0.2957
ko00920	Sulfur metabolism	0.0009	0.0221	0.3393	0.4614
ko05417	Lipid and atherosclerosis	0.0011	0.0241	0.1094	0.0969
ko03430	Mismatch repair	0.0012	0.0244	0.7326	0.6045
ko04112	Cell cycle - Caulobacter	0.0012	0.0249	0.4678	0.3924
ko03010	Ribosome	0.0015	0.0262	2.2627	1.844
ko00540	Lipopolysaccharide biosynthesis	0.0016	0.0262	0.5747	0.3855
ko00240	Pyrimidine metabolism	0.0016	0.0262	1.1548	0.9866
ko00621	Dioxin degradation	0.0015	0.0262	0.0341	0.0505
ko00350	Tyrosine metabolism	0.0016	0.0262	0.1601	0.2304
ko01240	Biosynthesis of cofactors	0.002	0.0267	3.6015	3.316
ko03030	DNA replication	0.002	0.0267	0.6094	0.5193
ko00710	Carbon fixation in photosynthetic organisms	0.0019	0.0267	0.4655	0.4045
ko04928	Parathyroid hormone synthesis, secretion and action	0.0019	0.0267	0.0005	0.0004
ko00622	Xylene degradation	0.0018	0.0267	0.0541	0.0797
ko00790	Folate biosynthesis	0.0021	0.0267	0.6014	0.5345
ko00071	Fatty acid degradation	0.0021	0.0267	0.2031	0.4004
ko03420	Nucleotide excision repair	0.0024	0.0271	0.3322	0.278
ko00460	Cyanoamino acid metabolism	0.0024	0.0271	0.152	0.143
ko01220	Degradation of aromatic compounds	0.0024	0.0271	0.1713	0.2926
ko00362	Benzoate degradation	0.0024	0.0271	0.2048	0.3826
ko03020	RNA polymerase	0.0026	0.0282	0.1666	0.1419
ko01232	Nucleotide metabolism	0.0041	0.0284	1.3013	1.1492
ko03440	Homologous recombination	0.003	0.0284	0.8669	0.7412
ko03060	Protein export	0.003	0.0284	0.5741	0.496
ko00195	Photosynthesis	0.0036	0.0284	0.3284	0.2773
ko00730	Thiamine metabolism	0.0034	0.0284	0.3919	0.3431
ko01502	Vancomycin resistance	0.0036	0.0284	0.2494	0.2112
ko00900	Terpenoid backbone biosynthesis	0.0043	0.0284	0.439	0.4012
ko00750	Vitamin B6 metabolism	0.0036	0.0284	0.1679	0.16
ko04914	Progesterone-mediated oocyte maturation	0.0046	0.0284	0.0238	0.0184

ko04612	Antigen processing and presentation	0.0046	0.0284	0.0238	0.0184
ko04657	IL-17 signaling pathway	0.0046	0.0284	0.0238	0.0184
ko04659	Th17 cell differentiation	0.0046	0.0284	0.0238	0.0184
ko05215	Prostate cancer	0.0046	0.0284	0.0238	0.0184
ko04915	Estrogen signaling pathway	0.0046	0.0284	0.0238	0.0184
ko03008	Ribosome biogenesis in eukaryotes	0.0038	0.0284	0.056	0.0513
ko00945	Stilbenoid, diarylheptanoid and gingerol biosynthesis	0.0032	0.0284	0.008	0.0041
ko00941	Flavonoid biosynthesis	0.0032	0.0284	0.008	0.0041
ko04137	Mitophagy - animal	0.0028	0.0284	2E-06	1E-06
ko05219	Bladder cancer	0.0036	0.0284	0.0043	0.0085
ko00624	Polycyclic aromatic hydrocarbon degradation	0.0041	0.0284	0.0039	0.0124
ko00642	Ethylbenzene degradation	0.0046	0.0284	0.0117	0.0289
ko00592	alpha-Linolenic acid metabolism	0.0043	0.0284	0.0169	0.0371
ko00623	Toluene degradation	0.0032	0.0284	0.0174	0.0382
ko00980	Metabolism of xenobiotics by cytochrome P450	0.0046	0.0284	0.0477	0.0747
ko00982	Drug metabolism - cytochrome P450	0.0043	0.0284	0.0486	0.0827
ko01040	Biosynthesis of unsaturated fatty acids	0.003	0.0284	0.0267	0.0643
ko00281	Geraniol degradation	0.0038	0.0284	0.0368	0.1246
ko00630	Glyoxylate and dicarboxylate metabolism	0.0038	0.0284	0.6078	0.7122
ko04611	Platelet activation	0.0049	0.0298	0.0005	0.0003
ko00550	Peptidoglycan biosynthesis	0.0067	0.0317	0.8066	0.6947
ko00300	Lysine biosynthesis	0.0063	0.0317	0.4997	0.4449
ko00030	Pentose phosphate pathway	0.0067	0.0317	0.6736	0.6193
ko00680	Methane metabolism	0.0063	0.0317	0.6232	0.5861
ko04066	HIF-1 signaling pathway	0.0067	0.0317	0.2161	0.1846
ko03410	Base excision repair	0.0067	0.0317	0.374	0.3489
ko04138	Autophagy - yeast	0.0056	0.0317	0.0045	0.0079
ko00565	Ether lipid metabolism	0.0063	0.0317	0.0067	0.0117
ko00965	Betalain biosynthesis	0.0059	0.0317	0.0063	0.0117
ko00120	Primary bile acid biosynthesis	0.0059	0.0317	0.0035	0.0094
ko04964	Proximal tubule bicarbonate reclamation	0.0059	0.0317	0.0148	0.0216
ko00830	Retinol metabolism	0.0059	0.0317	0.0287	0.04
ko00361	Chlorocyclohexane and chlorobenzene degradation	0.0056	0.0317	0.0317	0.0568
ko00660	C5-Branched dibasic acid metabolism	0.0067	0.0317	0.1959	0.2243
ko04975	Fat digestion and absorption	0.0059	0.0317	0.0319	0.0631
ko00310	Lysine degradation	0.0063	0.0317	0.1964	0.3275
ko00360	Phenylalanine metabolism	0.0056	0.0317	0.1845	0.3334
ko00280	Valine, leucine and isoleucine degradation	0.0063	0.0317	0.2836	0.4995
ko04974	Protein digestion and absorption	0.0071	0.0332	0.0189	0.0114
ko00640	Propanoate metabolism	0.0075	0.0348	0.5194	0.6113
ko00365	Furfural degradation	0.008	0.0365	0.0015	0.0011
ko00930	Caprolactam degradation	0.0084	0.0379	0.0214	0.0904
ko01120	Microbial metabolism in diverse environments	0.0084	0.0379	4.0735	4.4852
ko00430	Taurine and hypotaurine metabolism	0.0089	0.0388	0.132	0.1238
ko00253	Tetracycline biosynthesis	0.0089	0.0388	7E-05	0.0003
ko00290	Valine, leucine and isoleucine biosynthesis	0.0089	0.0388	0.3132	0.355
ko04621	NOD-like receptor signaling pathway	0.0106	0.0451	0.1063	0.0882

ko00562	Inositol phosphate metabolism	0.0106	0.0451	0.0897	0.1079
ko00010	Glycolysis / Gluconeogenesis	0.0113	0.0468	0.9944	0.9251
ko01051	Biosynthesis of ansamycins	0.0113	0.0468	0.0619	0.0545
ko00720	Carbon fixation pathways in prokaryotes	0.0126	0.0513	0.8088	0.745
ko05130	Pathogenic Escherichia coli infection	0.0126	0.0513	0.0639	0.0561
ko03240	Viral replication	0.0131	0.0526	7E-07	0
ko00903	Limonene and pinene degradation	0.0133	0.0526	0.0292	0.1135
ko00380	Tryptophan metabolism	0.0133	0.0526	0.1708	0.3209
ko03450	Non-homologous end-joining	0.0141	0.054	0.0063	0.0141
ko00625	Chloroalkane and chloroalkene degradation	0.0141	0.054	0.0613	0.1027
ko01212	Fatty acid metabolism	0.0141	0.054	0.5625	0.7231
ko00520	Amino sugar and nucleotide sugar metabolism	0.0149	0.0559	1.0831	0.9718
ko00062	Fatty acid elongation	0.0149	0.0559	0.0005	0.0014
ko03320	PPAR signaling pathway	0.0157	0.0585	0.1163	0.1625
ko05134	Legionellosis	0.0175	0.0646	0.1347	0.1227
ko01057	Biosynthesis of type II polyketide products	0.0195	0.0706	0.0013	0.0011
ko00364	Fluorobenzoate degradation	0.0195	0.0706	0.016	0.0307
ko04936	Alcoholic liver disease	0.0205	0.0724	0.0304	0.0904
ko00650	Butanoate metabolism	0.0205	0.0724	0.5651	0.6721
ko02010	ABC transporters	0.0205	0.0724	2.5661	2.8496
ko00270	Cysteine and methionine metabolism	0.0216	0.075	0.9903	0.9083
ko00250	Alanine, aspartate and glutamate metabolism	0.0216	0.075	0.7176	0.7023
ko04070	Phosphatidylinositol signaling system	0.0228	0.0776	0.0838	0.0808
ko00410	beta-Alanine metabolism	0.0228	0.0776	0.1113	0.2031
ko04115	p53 signaling pathway	0.024	0.0811	0.0046	0.0077
ko05165	Human papillomavirus infection	0.0252	0.0839	0.0424	0.0386
ko00643	Styrene degradation	0.0252	0.0839	0.0432	0.0703
ko01100	Metabolic pathways	0.0266	0.0855	17.516	17.279
ko04978	Mineral absorption	0.0266	0.0855	0.0665	0.0593
ko05110	Vibrio cholerae infection	0.0266	0.0855	0.0029	0.0034
ko04113	Meiosis - yeast	0.0266	0.0855	0.0199	0.0349
ko00564	Glycerophospholipid metabolism	0.0279	0.0885	0.4538	0.4314
ko04930	Type II diabetes mellitus	0.0279	0.0885	0.0429	0.039
ko05203	Viral carcinogenesis	0.0294	0.0923	0.0419	0.0383
ko00600	Sphingolipid metabolism	0.0325	0.0952	0.1243	0.1036
ko00603	Glycosphingolipid biosynthesis - globo and isoglobo se	0.0309	0.0952	0.06	0.0492
ko00563	Glycosylphosphatidylinositol (GPI)-anchor biosynthesis	0.0322	0.0952	6E-05	1E-05
ko05142	Chagas disease	0.0309	0.0952	0.0079	0.0109
ko05034	Alcoholism	0.0325	0.0952	0.001	0.0083
ko04726	Serotonergic synapse	0.0325	0.0952	0.001	0.0083
ko05030	Cocaine addiction	0.0325	0.0952	0.001	0.0083
ko05031	Amphetamine addiction	0.0325	0.0952	0.001	0.0083
ko04714	Thermogenesis	0.0325	0.0952	0.1021	0.1124
ko00230	Purine metabolism	0.0341	0.0986	1.5236	1.4302
ko00627	Aminobenzoate degradation	0.0341	0.0986	0.0714	0.1449
ko04977	Vitamin digestion and absorption	0.0354	0.1016	8E-06	3E-06
ko00470	D-Amino acid metabolism	0.0358	0.102	0.3167	0.2952

ko04120	Ubiquitin mediated proteolysis	0.0363	0.1027	8E-06	0.0001
ko00534	Glycosaminoglycan biosynthesis - heparan sulfate / he|	0.0368	0.1035	7E-06	3E-06
ko04713	Circadian entrainment	0.0414	0.1139	2E-07	2E-06
ko04740	Olfactory transduction	0.0414	0.1139	2E-07	2E-06
ko00626	Naphthalene degradation	0.0414	0.1139	0.0394	0.0539
ko01054	Nonribosomal peptide structures	0.0455	0.1235	0.0021	0.0016
ko00960	Tropane, piperidine and pyridine alkaloid biosynthesis	0.0455	0.1235	0.0751	0.0803
ko00670	One carbon pool by folate	0.0477	0.1251	0.4239	0.3821
ko00513	Various types of N-glycan biosynthesis	0.0477	0.1251	0.0416	0.0318
ko00604	Glycosphingolipid biosynthesis - ganglio series	0.0477	0.1251	0.0416	0.0317
ko00332	Carbapenem biosynthesis	0.0477	0.1251	0.0401	0.047
ko04146	Peroxisome	0.0477	0.1251	0.1808	0.1996
ko02025	Biofilm formation - Pseudomonas aeruginosa	0.0499	0.1302	0.2505	0.3464

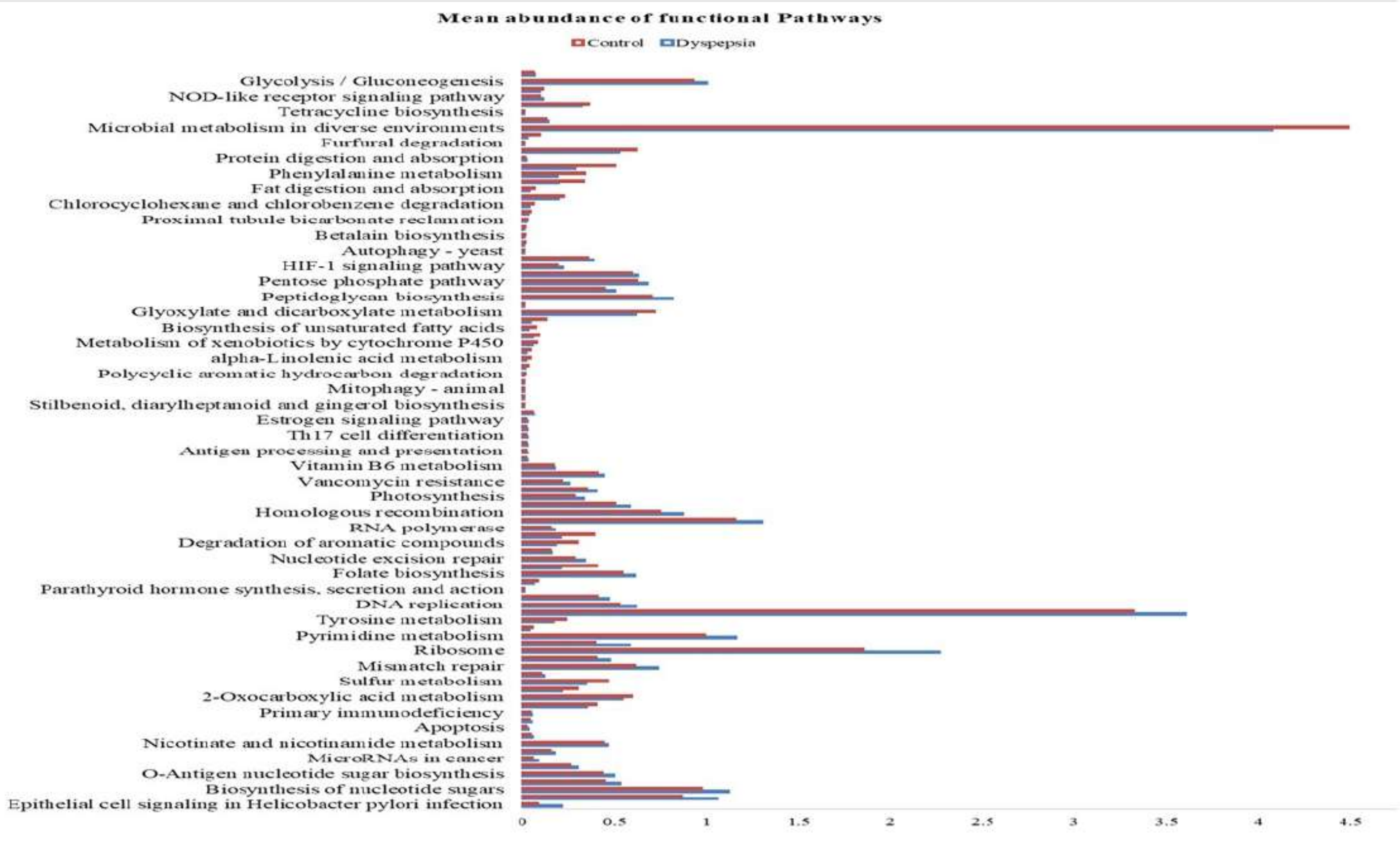

Supplementary Figure 7: Mean abundance of predicted functional pathways across dyspepsia and control groups
