# Supplementary material for: Analysis of gastric mucosa associated microbiota in functional dyspepsia using 16S rRNA gene next-generation sequencing
Source: BMC Microbiol. 2025 Jun 26;25:368. doi: 10.1186/s12866-025-04095-0 (PMC12199507; doi:10.1186/s12866-025-04095-0)
Supplement: Supplementary file 2 — Supplementary Material 2. [file 12866_2025_4095_MOESM2_ESM.pdf]

Accession	BioProject	BioSample	Status	Release Date
SRR32438639	PRJNA1226534	SAMN46941449	Released	2/22/2025
SRR32438636	PRJNA1226534	SAMN46941447	Released	2/22/2025
SRR32438633	PRJNA1226534	SAMN46941440	Released	2/22/2025
SRR32438519	PRJNA1226534	SAMN46941444	Released	2/22/2025
SRR32438640	PRJNA1226534	SAMN46941438	Released	2/22/2025
SRR32438637	PRJNA1226534	SAMN46941443	Released	2/22/2025
SRR32438634	PRJNA1226534	SAMN46941445	Released	2/22/2025
SRR32438520	PRJNA1226534	SAMN46941441	Released	2/22/2025
SRR32438517	PRJNA1226534	SAMN46941439	Released	2/22/2025
SRR32438638	PRJNA1226534	SAMN46941442	Released	2/22/2025
SRR32438635	PRJNA1226534	SAMN46941437	Released	2/22/2025
SRR32438632	PRJNA1226534	SAMN46941446	Released	2/22/2025
SRR32438518	PRJNA1226534	SAMN46941448	Released	2/22/2025
SRR32426797	PRJNA1226534	SAMN46941426	Released	2/21/2025
SRR32427872	PRJNA1226534	SAMN46941436	Released	2/21/2025
SRR32427640	PRJNA1226534	SAMN46941433	Released	2/21/2025
SRR32427637	PRJNA1226534	SAMN46941431	Released	2/21/2025
SRR32427638	PRJNA1226534	SAMN46941432	Released	2/21/2025
SRR32427642	PRJNA1226534	SAMN46941434	Released	2/21/2025
SRR32427636	PRJNA1226534	SAMN46941435	Released	2/21/2025
SRR32427633	PRJNA1226534	SAMN46941429	Released	2/21/2025
SRR32427627	PRJNA1226534	SAMN46941430	Released	2/21/2025
SRR32427628	PRJNA1226534	SAMN46941428	Released	2/21/2025
SRR32427569	PRJNA1226534	SAMN46941427	Released	2/21/2025
SRR32426585	PRJNA1226534	SAMN46941414	Released	2/21/2025
SRR32426799	PRJNA1226534	SAMN46941425	Released	2/21/2025
SRR32426794	PRJNA1226534	SAMN46941424	Released	2/21/2025
SRR32426632	PRJNA1226534	SAMN46941423	Released	2/21/2025
SRR32426629	PRJNA1226534	SAMN46941422	Released	2/21/2025
SRR32426617	PRJNA1226534	SAMN46941419	Released	2/21/2025
SRR32426604	PRJNA1226534	SAMN46941418	Released	2/21/2025
SRR32426598	PRJNA1226534	SAMN46941417	Released	2/21/2025
SRR32426593	PRJNA1226534	SAMN46941416	Released	2/21/2025
SRR32426592	PRJNA1226534	SAMN46941415	Released	2/21/2025
SRR32426588	PRJNA1226534	SAMN46941413	Released	2/21/2025
SRR32426581	PRJNA1226534	SAMN46941412	Released	2/21/2025
SRR32426560	PRJNA1226534	SAMN46941411	Released	2/21/2025
SRR32426558	PRJNA1226534	SAMN46941410	Released	2/21/2025
SRR32426410	PRJNA1226534	SAMN46941408	Released	2/21/2025
SRR32426409	PRJNA1226534	SAMN46941409	Released	2/21/2025
	PRJNA1226534	SAMN46941459	Processing	2/22/2025
	PRJNA1226534	SAMN46941453	Processing	2/22/2025
	PRJNA1226534	SAMN46941456	Processing	2/22/2025
	PRJNA1226534	SAMN46941458	Processing	2/22/2025
	PRJNA1226534	SAMN46941457	Processing	2/22/2025
	PRJNA1226534	SAMN46941451	Processing	2/22/2025
	PRJNA1226534	SAMN46941452	Processing	2/22/2025
	PRJNA1226534	SAMN46941450	Processing	2/22/2025
	PRJNA1226534	SAMN46941455	Processing	2/22/2025

PRJNA1226534	SAMN46941454	Processing	2/22/2025
